# Prognostic nomograms for gastric carcinoma after D2 + total gastrectomy to assist decision-making for postoperative treatment: based on Lasso regression

**DOI:** 10.1186/s12957-023-03097-4

**Published:** 2023-07-20

**Authors:** Yifan Li, Min Bai, Yuye Gao

**Affiliations:** 1 Second Department of General Surgery, Shanxi Province Carcinoma Hospital, Shanxi Hospital Affiliated to Carcinoma Hospital, Chinese Academy of Medical Sciences, Carcinoma Hospital Affiliated to Shanxi Medical University, Taiyuan, Shanxi 030013 People’s Republic of China; 2 Department of Hematopathology, Shanxi Province Carcinoma Hospital, Shanxi Hospital Affiliated to Carcinoma Hospital, Chinese Academy of Medical Sciences, Carcinoma Hospital Affiliated to Shanxi Medical University, Taiyuan, Shanxi 030013 People’s Republic of China; 3 Department of Gastrointestinal Surgery, Key Laboratory of Carcinogenesis and Translational Research (Ministry of Education/Beijing), Peking University Cancer Hospital and Institute, No. 52 Fu Cheng Road, Hai Dian District, Beijing, 100142 China

**Keywords:** Gastric carcinoma, Total gastrectomy, Overall survival, Progress-free survival

## Abstract

**Objective:**

This study aimed to establish novel nomograms that could be used to predict the prognosis of gastric carcinoma patients who underwent D2 + total gastrectomy on overall survival (OS) and progression-free survival (PFS).

**Methods:**

Lasso regression was employed to construct the nomograms. The internal validation process included bootstrapping, which was used to test the accuracy of the predictions. The calibration curve was then used to demonstrate the accuracy and consistency of the predictions. In addition, the Harrell’s Concordance index (C-index) and time-dependent receiver operating characteristic (t-ROC) curves were used to evaluate the discriminative abilities of the new nomograms and to compare its performance with the 8th edition of AJCC-TNM staging. Furthermore, decision curve analysis (DCA) was performed to assess the clinical application of our model. Finally, the prognostic risk stratification of gastric cancer was conducted with X-tile software, and the nomograms were converted into a risk-stratifying prognosis model.

**Results:**

LASSO regression analysis identified pT stage, the number of positive lymph nodes, vascular invasion, neural invasion, the maximum diameter of tumor, the Clavien–Dindo classification for complication, and Ki67 as independent risk factors for OS and pT stage, the number of positive lymph nodes, neural invasion, and the maximum diameter of tumor for PFS. The C-index of OS nomogram was 0.719 (95% CI: 0.690–0.748), which was superior to the 8th edition of AJCC-TNM staging (0.704, 95%CI: 0.623–0.783). The C-index of PFS nomogram was 0.694 (95% CI: 0.654–0.713), which was also better than that of the 8th edition of AJCC-TNM staging (0.685, 95% CI: 0.635–0.751). The calibration curves, t-ROC curves, and DCA of the two nomogram models showed that the prediction ability of the two nomogram models was outstanding. The statistical difference in the prognosis between the low- and high-risk groups further suggested that our model had an excellent risk stratification performance.

**Conclusion:**

We reported the first risk stratification and nomogram for gastric carcinoma patients with total gastrectomy in Chinese population. Our model could potentially be used to guide treatment selections for the low- and high-risk patients to avoid delayed treatment or unnecessary overtreatment.

## Background

Gastric carcinoma is one of the most common malignant tumors worldwide, and it mainly occurs in the northwest and eastern coastal China [1–3]. There is an increasing incidence of carcinoma in the upper third of the stomach and gastroesophageal junction in both Western and Eastern countries [4, 5], and total gastrectomy is a standard treatment for these patients [6, 7]. However, to ensure better surgical margins and a more radical lymphadenectomy, total gastrectomy tends to result in postoperative malnutrition, severe complications, and even major morbidities which can be serious and fatal in short periods after surgery [8, 9]. Total gastrectomy is among the most invasive gastrointestinal procedures and is known to carry substantial surgical risks. Although Roux-en-Y has long been the most common type of reconstruction after total gastrectomy worldwide, it cannot restore the loss of reservoir capacity caused by total gastrectomy, which is the major reason for the deteriorated quality of life and malnutrition of patients [10, 11]. In consistent with this, despite the 1-, 3-, and 5-year survival rate of all sample was 91.4% (1055/1099), 59.3% (604/1019), and 36.9% (372/1008), respectively, the 1-, 3-, and 5-year survival rate after total gastrectomy was only 89.6% (508/567), 49.4% (271/549), and 27.3% (151/553), respectively, indicating that the survival status of patients with total gastrectomy was much worse than overall patients. Therefore, construction models to predict the prognosis of patients who have undergone D2 + total gastrectomy are critical for the health management of gastric carcinoma patients. It will be significant to perform risk stratification of patients with total gastrectomy to distinguish patients between high- and low-probability of recurrence. Hence, our risk prediction model might provide accurate guidance for the postoperative treatment of gastric carcinoma patients.

## Methods

### Patient enrollment

Between May 2002 and December 2020, 1708 patients who had undergone radical gastric surgery for gastric carcinoma were retrospectively analyzed. To be included in this study, the patients should be histologically diagnosed as gastric carcinoma and have received curative surgery (R0-R1). Finally, a total of 944 patients who underwent D2 + total gastrectomy were enrolled in this study and were randomly divided into the training cohort (660 cases) and the validation cohort (284 cases) at a ratio of 7:3 [12].

**Fig. 1:**
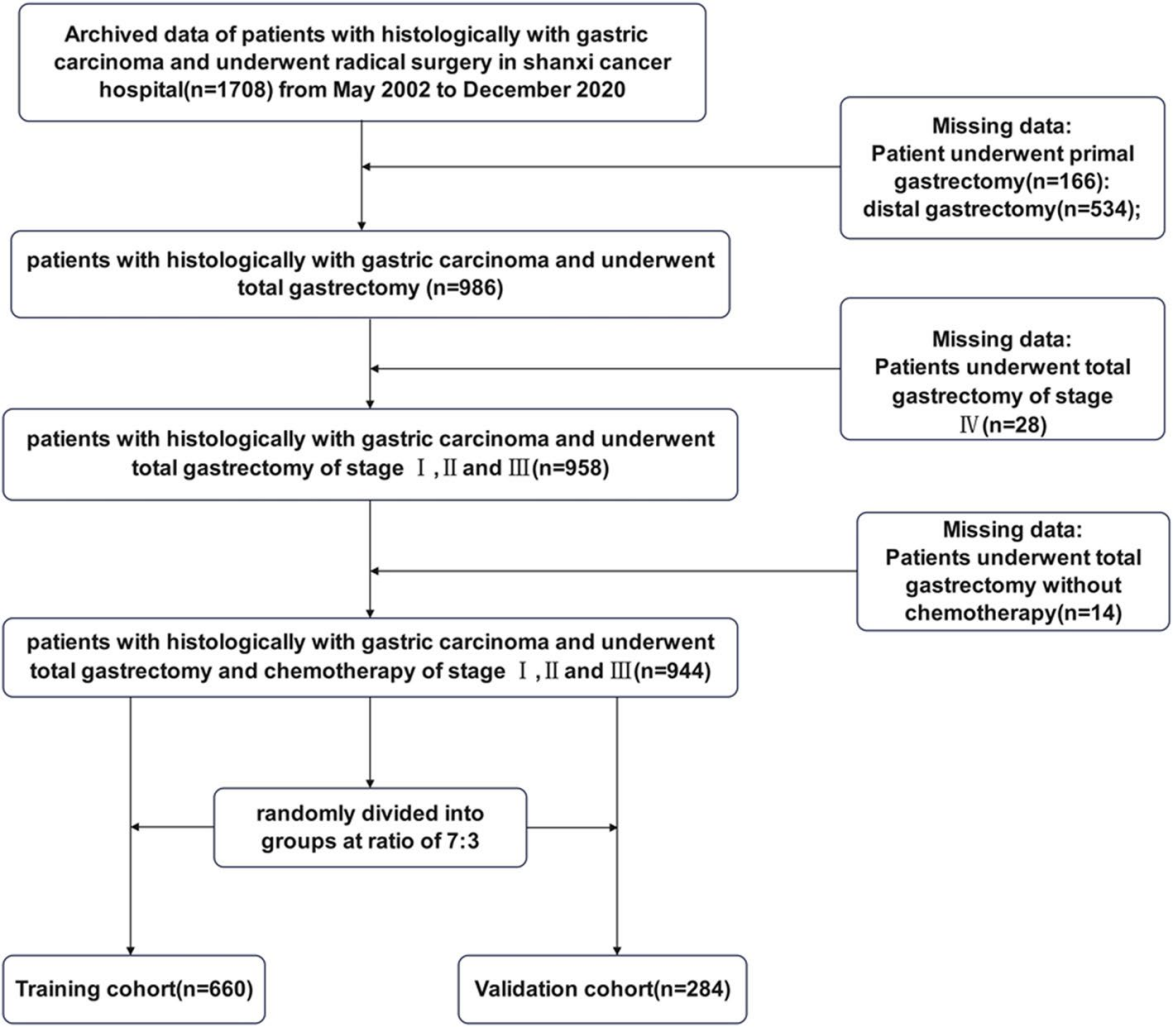
The flowchart of study population enrolment in the training and validation cohort of gastric cancer

The specific inclusion criteria were (1) gastric cancer confirmed by histological pathology; (2) had curative total gastrectomy for stages I, II, and III; (3) complete clinicopathological and follow-up data available; (4) no severe organ damage after surgery; and (5) no other malignant tumors. The exclusion criteria were (1) with other systemic tumors; (2) missing or with incomplete clinical data; (3) had palliative surgery or bypass surgery; (4) pathological classification confirmed as non-gastric cancer; (5) had distal gastrectomy or primal gastrectomy; and (6) had total gastrectomy of stage IV. Due to the retrospective nature of the study, informed written consent form could not be obtained from patients; however, the Ethics Committee of Shanxi Cancer Hospital reviewed and approved the study protocol. Furthermore, in accordance with the Declaration of Helsinki, all patient data were anonymous and strictly confidential [12]. A detailed research flowchart was shown in Fig. 1.

### Clinicopathological data of patients

The following clinical information of each patient was collected: gender, age at surgery, vascular invasion, neural invasion, pT stage, number of positive lymph nodes, TNM stage (according to the 8th edition of the American Joint Committee on Cancer Staging), Lauren classification, maximum diameter of tumor, omentum metastasis, surgical margin, multiple organ resection, histological classification, Clavien–Dindo classification for complication, AE1/AE3, Ki67 (%), CK7, CK20, CDX-2, SATB-2, SYN, CGA, CD56, MLH1, PMS2, MSH2, MSH6, overall survival (OS), and progression-free survival (PFS). OS was calculated based on the time the patient died or the last follow-up time [12], while PFS was defined as the period after surgery to the diagnosis of metastasis.

We determined the follow-up time by analyzing medical records from both the hospital and oncologists during the patient's stay. The follow-up time was calculated based on the patient’s last hospital visit or the last contact to doctor.

### Statistical analysis

Descriptive statistical analysis was expressed as an absolute percentage of a categorical variable, while the median of a continuous variable was derived as a median of its interquartile range. We presented OS and PFS using Kaplan–Meier curves. The Lasso regression method was applied to analyze and identify factors related to OS and PFS. The results were presented as hazard ratios, 95% CI, and *P* values. *P* < 0.05 was considered statistically significant. A variety of software was utilized to process data, including R software (version 4.1.2), SPSS 25.0, and GrandPad Prism 9.3.

### Nomogram construction and performance validation

The main goal of this study was to construct nomograms to predict 1-year, 3-year, 5-year OS, and PFS of patients with gastric cancer after D2 + total gastrectomy. The parameters used for the model construction were derived from the Lasso regression analysis. The performance of the models was then computed through 10,000 repetitions. We tested the accuracy of the nomograms by discrimination and calibration in both the internal and external validation cohorts. Furthermore, the discriminative capability of the established model was evaluated according to Harrell’s concordance index (C-index), the time-dependent receiver operating characteristic curve (t-ROC), and area under the ROC curve (AUC). Calibration curves were constructed to compare the predicted OS and PFS with the observed OS and PFS using a bootstrap approach with 1000 resamples, while a DCA assessed the clinical implication of the model [1–3, 13]. Harrell’s concordance index (C-index), which was the agreement between the predictions and the observations. In terms of the C-index, the value ranged from 0.5 to 1.0. The value 0.5 indicated the random events of correctly differentiating between the outcomes by the model, while the value 1.0 indicated perfect discrimination accuracy. To verify the prognosis-distinguishment ability of the nomogram scoring model in gastric cancer patients, the total score of each patient in the development cohort was calculated. The best cut-off values of the total score were determined using the X-tile software with adjustment. Patients in the development and validation cohorts were stratified into high- and low-risk groups. In accordance with the total score, the cut-off values of the total simplified score were determined by the X-tile software with adjustment. Patients in the development cohort were divided into high- and low-risk groups, and the same classification algorithm was used in the validation cohort. The Log-rank test with pairwise comparisons in the Kaplan**–**Meier survival analysis was used to compare the survival times of different risk groups. Data were presented as the mean ± standard deviation (SD), median [interquartile range, IQR], or number (%). All statistical analyses were performed using SPSS (version 25.0, SPSS Inc.) and R software (version 4.1.2). A two-tailed *p* value < 0.05 was considered statistically significant.

## Results

### Patient clinical characteristics

According to the inclusion and exclusion criteria, 944 eligible patients with gastric carcinoma who underwent D2 + total gastrectomy were enrolled in this study, including 660 in the training cohort and 284 in the validation cohort. The flowchart of this study was shown in Fig. 1. In the training cohort, 280 (42.4%) of the 660 patients died, while 142 (46.5%) of the 284 patients in the validation cohort died. There was no significant difference in the clinicopathological features of patients between these two groups (Table 1).

**Table 1:** Baseline clinical features

Variables	Training cohort	Validation cohort
	**Mean ± SD/No. (%)**	**Mean ± SD/No. (%)**
Gender		
Male	548 (83.0%)	152(53.5%)
Female	112 (17.0%)	132 (46.5%)
Age (year)	58.95 ± 9.68	60.71 ± 9.19
Depth of tumor invasion		
T1	52 (7.9%)	29 (10.2%)
T2	22 (3.3%)	10 (3.5%)
T3	217 (32.9%)	86 (30.3%)
T4	369 (55.9%)	159 (56.0%)
Number of positive lymph nodes		
0	169 (25.6%)	62( 22.9%)
1–2	137 (20.8%)	56 (19.7%)
3–6	95 (14.4%)	51 (18.0%)
> 7	259 (39.2%)	112 (39.4%)
TNM Stage		
I	63 (9.5%)	34 (12.0%)
II	179 (27.1%)	61 (21.5%)
III	418 (63.3%)	189 (66.5%)
Vascular invasion		
Negative	255 (38.6%)	106 (37.3%)
Positive	405 (61.4%)	178 (62.7%)
Neural invasion		
Negative	307 (46.5%)	111 (39.1%)
Positive	353 (53.5%)	173 (60.9%)
Lauren classification		
Intestinal	213 (32.3%)	93 (32.7%)
Diffuse	274 (41.5%)	116 (40.8%)
Mixed	173 (26.2%)	75 (26.4%)
Maximum diameter of Tumor (cm)		
< 6	339 (51.4%)	143 (50.4%)
≥ 6	321 (48.6%)	141 (49.6%)
Omentum metastasis		
Negative	635 (96.2%)	275 (96.8%)
Positive	25 (3.8%)	9 (3.2%)
Surgical margin		
Negative	618 (93.6%)	264 (93.0%)
Positive	42 (6.4%)	20 (7.0%)
Her-2		
( −)	394 (59.7%)	178 (62.7%)
( +)	219 (33.2%)	88 (31.0%)
(+ +)	35 (5.3%)	11 (3.9%)
(+ + +)	12 (1.8%)	7 (2.5%)
Multiple organ excision		
No	621 (94.1%)	269 (94.7%)
Yes	39 (5.9%)	15 (5.3%)
Clavien–Dindo classification for complication		
Grade I	472 (71.2%)	195 (68.7%)
Grade II	94 (14.2%)	48 (16.9%)
Grade II	82 (12.4%)	34 (12.0%)
Grade IV	4 (0.6%)	4 (1.4%)
Grade IV	8 (1.2%)	3 (1.1%)
Histological classification		
Adenocarcinoma	540 (81.8%)	238 (83.8%)
Others	120 (18.2%)	46 (16.2%)
AE1/AE3		
Negative	99 (15.0%)	51 (18.0%)
Positive	561 (85.0%)	233 (82.0%)
Ki67(%)	60.68 ± 23.94	61.32 ± 23.16
CK7		
Negative	341 (51.7%)	145 (51.1%)
Positive	319 (48.3%)	139 (48.9%)
CK20		
Negative	485 (73.5%)	205 (72.2%)
Positive	135 (26.5%)	79 (27.8%)
CDX-2		
Negative	349 (52.9%)	156 (54.9%)
Positive	311 (47.1%)	128 (45.1%)
SATB-2		
Negative	536 (81.2%)	235 (82.7%)
Positive	124 (18.8%)	49 (17.3%)
SYN		
Negative	473 (71.7%)	203 (71.5%)
Positive	187 (28.3%)	81 (28.5%)
CGA		
Negative	542 (82.1%)	232 (81.7%)
Positive	118 (17.9%)	52 (18.3%)
CD56		
Negative	407 (61.7%)	173 (60.9%)
Positive	253 (38.3%)	111 (39.1%)
MLH1		
Negative	71 (10.8%)	22 (7.7%)
Positive	589 (89.2%)	262 (92.3%)
PMS2		
Negative	158 (23.9%)	55 (19.4%)
Positive	502 (76.1%)	293 (80.6%)
MSH2		
Negative	60 (9.1%)	25 (8.8%)
Positive	600 (90.9%)	259 (91.2%)
MSH6		
Negative	67 (10.2%)	19 (6.7%)
Positive	593 (89.8%)	265 (93.3%)
Overall survival (months)		
	36.12 ± 21.85	35.75 ± 21.20
Progression-free survival (months)		
	30.58 ± 22.51	31.70 ± 22.05
Status		
Censored	380 (57.6%)	152 (53.5%)
Mortality`	280 (42.4%)	142 (46.5%)

### Development and validation of the prediction model for overall survival (OS)

**Fig. 2:**
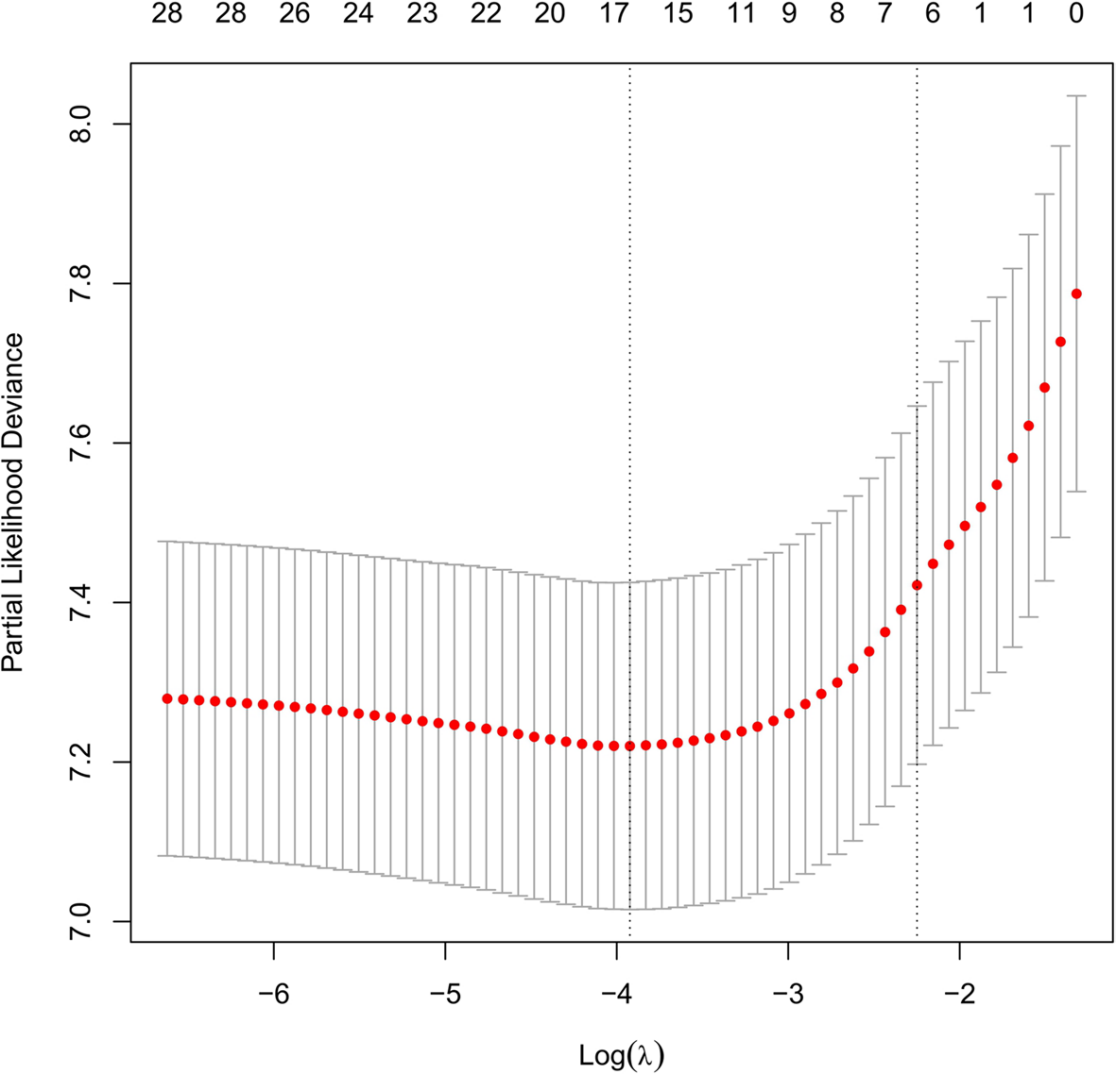
LASSO coefficient profiles of the 29 variables included in the model against the log lambda associated with overall survival (OS) of total gastrectomy

**Fig. 3:**
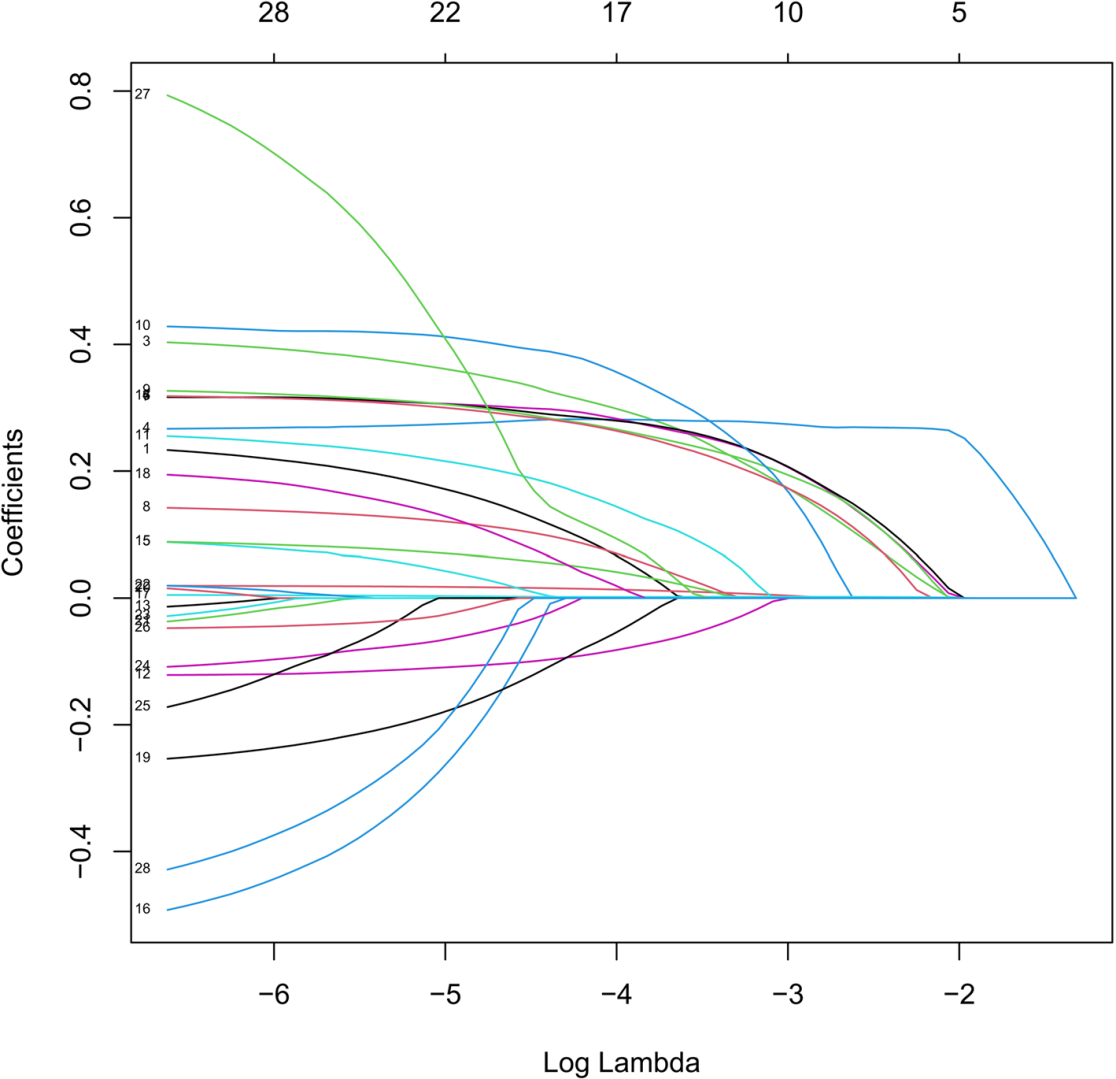
Relationship between the log lambda and the mean-squared error in the LASSO regression of overall survival (OS) of total gastrectomy

We performed a Lasso regression analysis to select the reliable variables to be used for the construction of predictive model for OS. Most of the covariate coefficients shrank to zero, and only 7 remaining nonzero parameters were selected as the independent prognostic factors of the model (Figs. 2 and 3). Among these factors, some have been known to be associated to the outcomes of patients with gastric cancer after D2 + total gastrectomy.

**Fig. 4:**
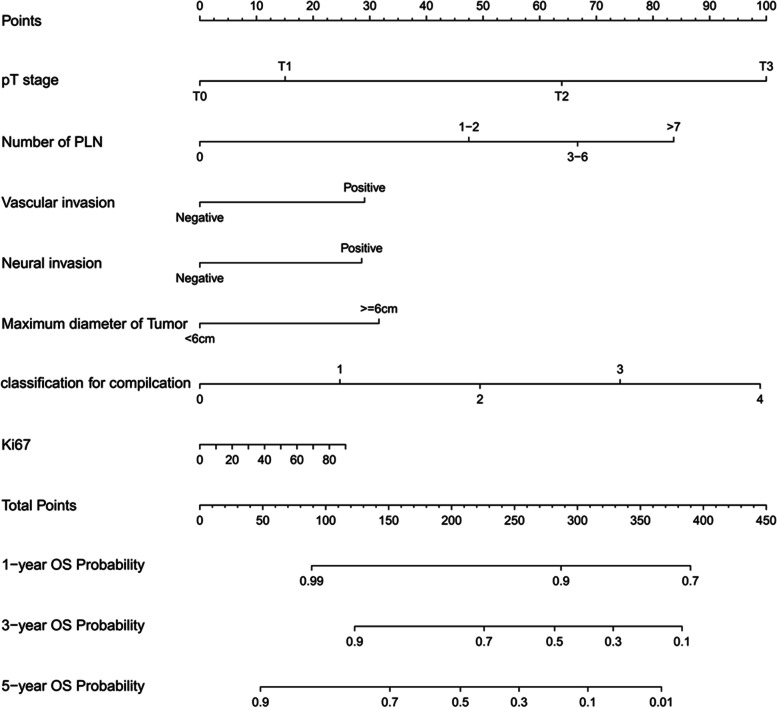
Nomogram model to predict 3-year and 5-year overall survival (OS) of total gastrectomy

Based on these diagnosis-related factors, we developed a prognostic nomogram for predicting the OS of gastric carcinoma patients (Fig. 4). In the nomogram plot, each variable was assigned a corresponding point according to its HR. Next, the total points were obtained by adding up the points for each variable and were positioned on the total point scale. This nomogram was used to predict the 1-, 3-, and 5-year overall survival (OS) of patients with gastric carcinoma who underwent D2 + total gastrectomy. The results showed that this nomogram model could predict the outcomes of gastric cancer patients (Fig. 4). In the training cohort, the C-index for OS was 0.719 (95% CI: 0.690–0.748). Importantly, when we compared this nomogram with the 8th edition of AJCC-TNM staging, the C-index of our nomogram was better than that of the 8th edition of AJCC-TNM staging (0.719 vs 0.704, 95% CI: 0.623–0.783).

### Validation of the predictive accuracy of the nomograms for OS

**Fig. 5:**
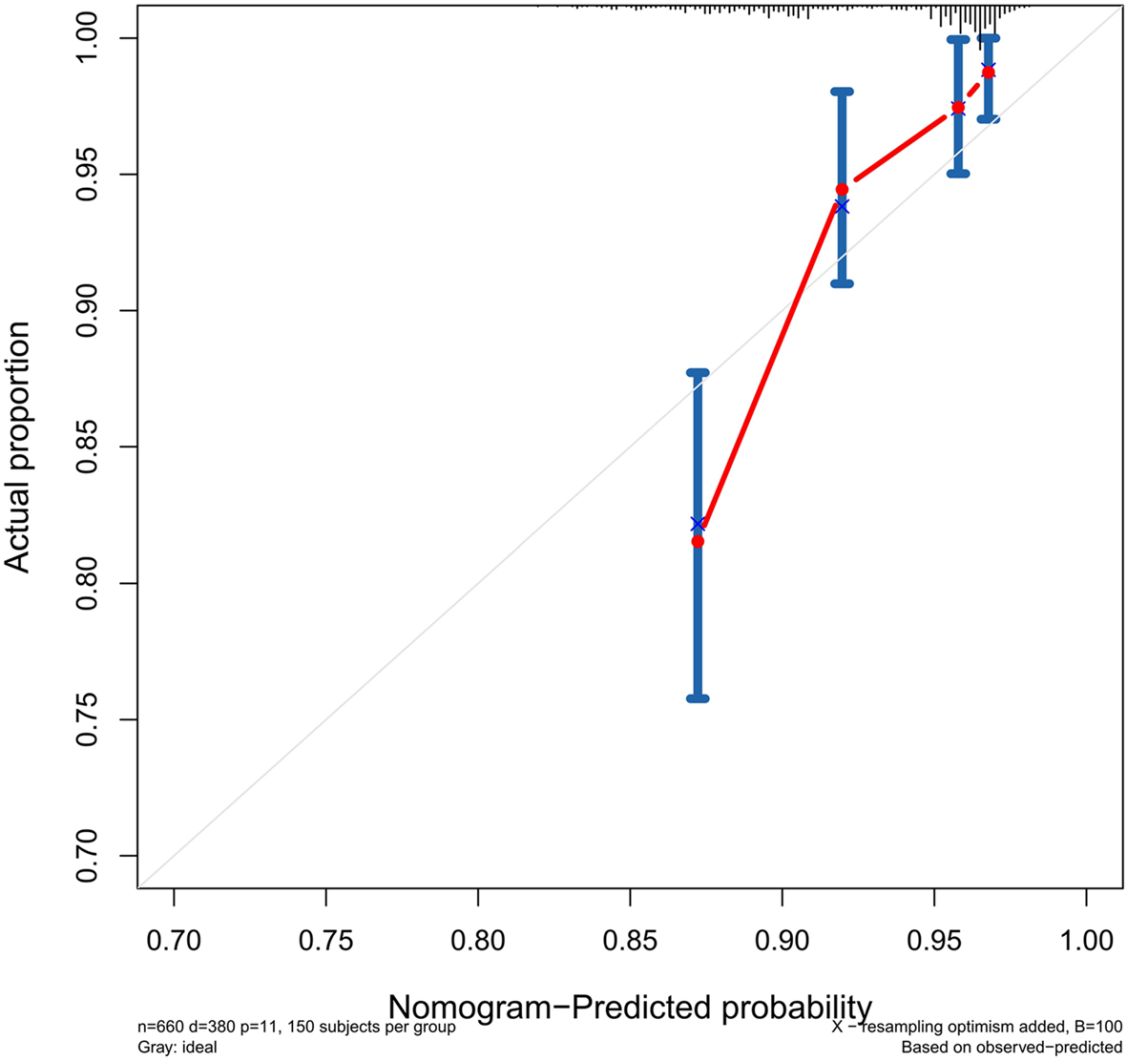
Calibration curves of internal validation to predict 1- year overall survival (OS) of total gastrectomy

**Fig. 6:**
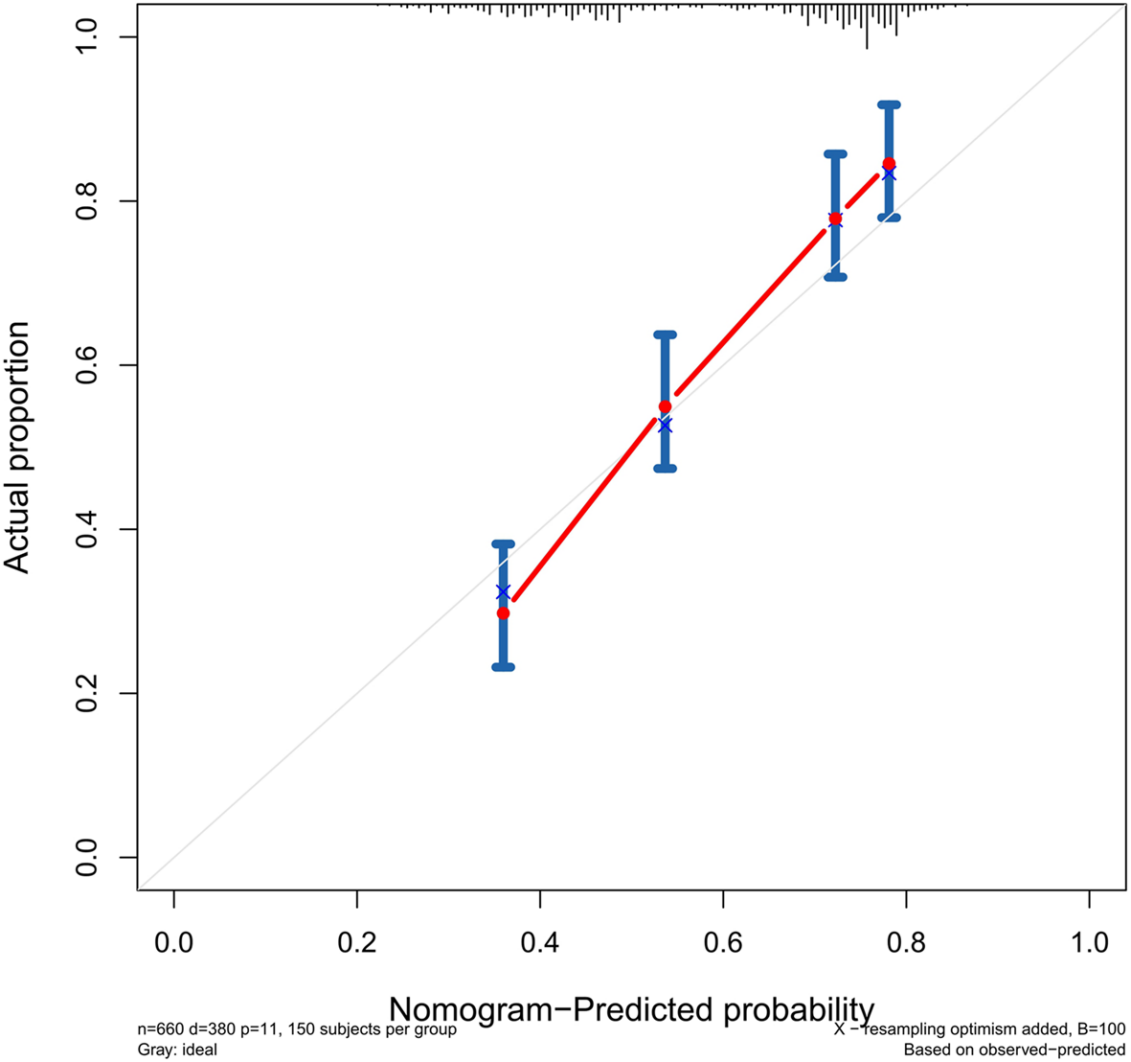
Calibration curves of internal validation to predict 3- year overall survival (OS) of total gastrectomy

**Fig. 7:**
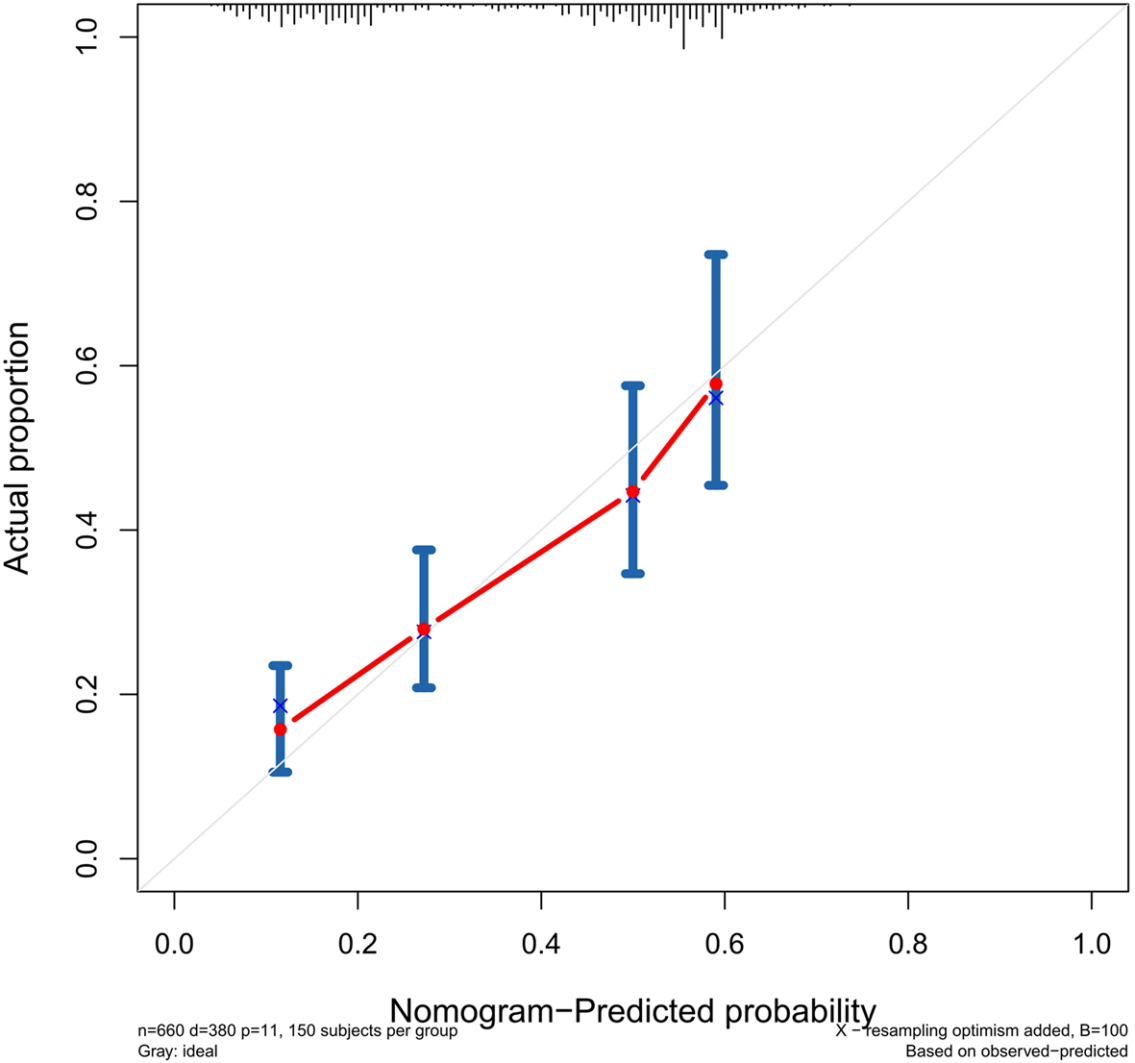
Calibration curves of internal validation to predict 5- year overall survival (OS) of total gastrectomy

**Fig. 8:**
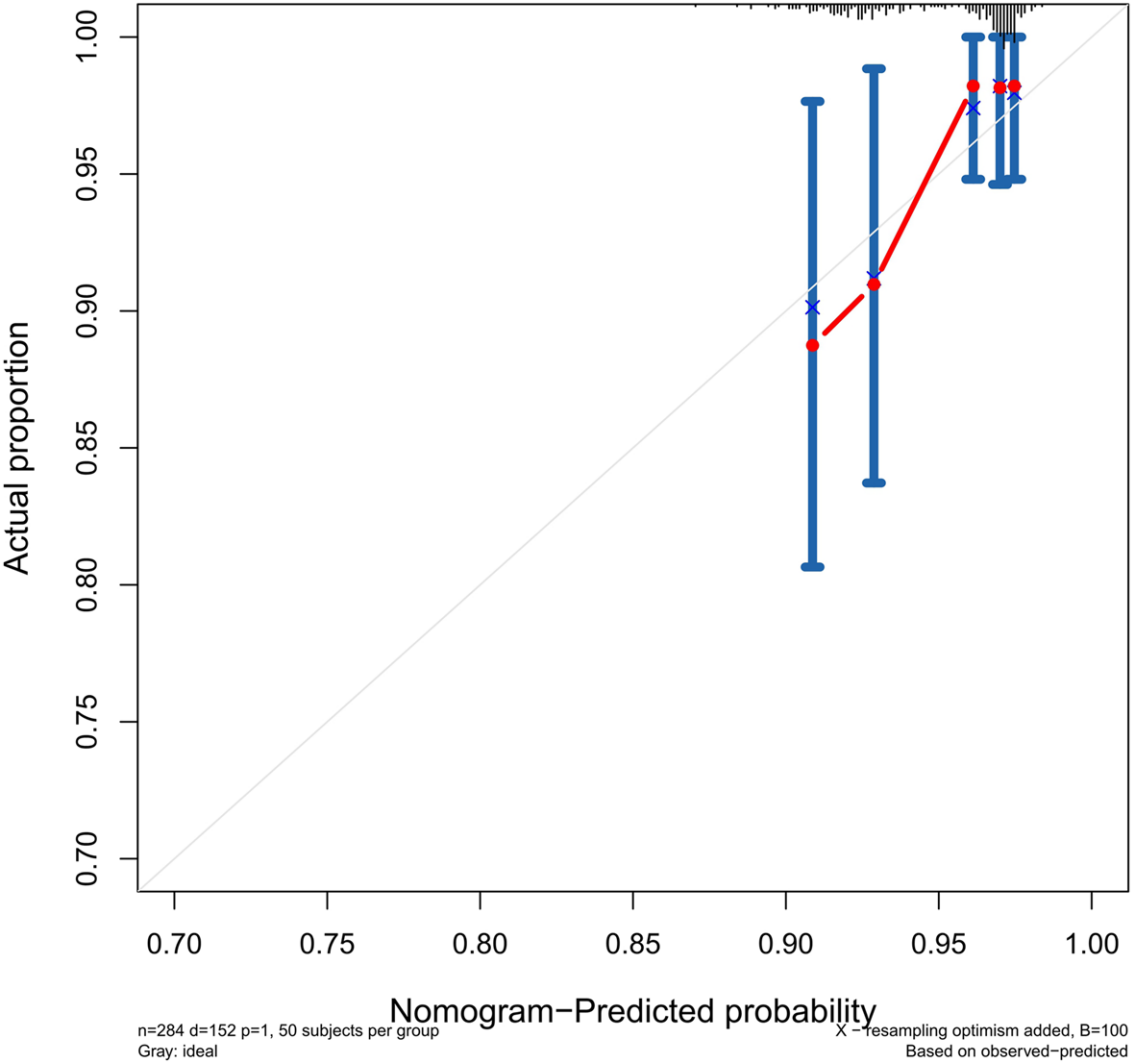
Calibration curves of external validation to predict 1- year OS of total gastrectomy

**Fig. 9:**
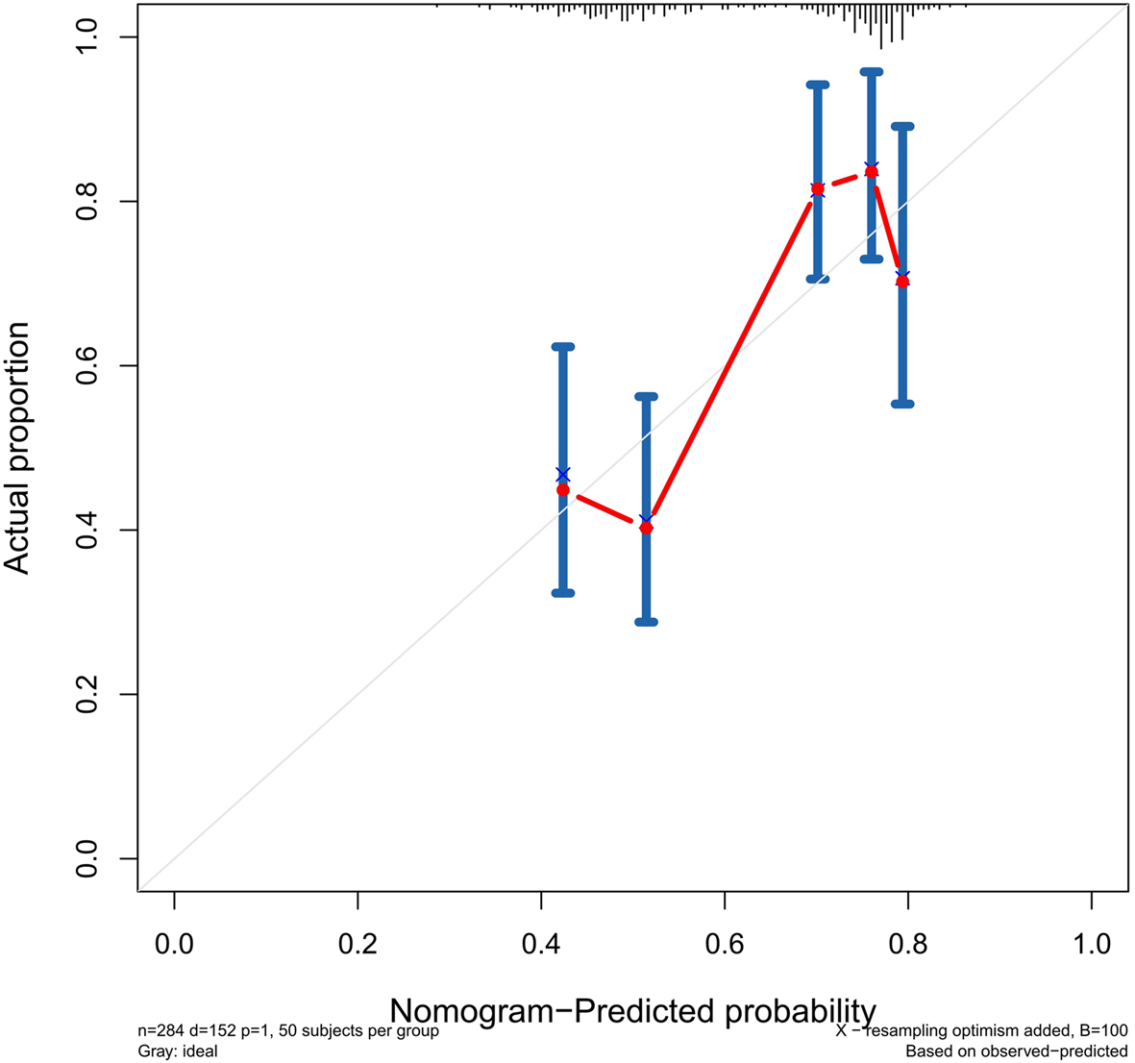
Calibration curves of external validation to predict 3- year overall survival (OS) of total gastrectomy

**Fig. 10:**
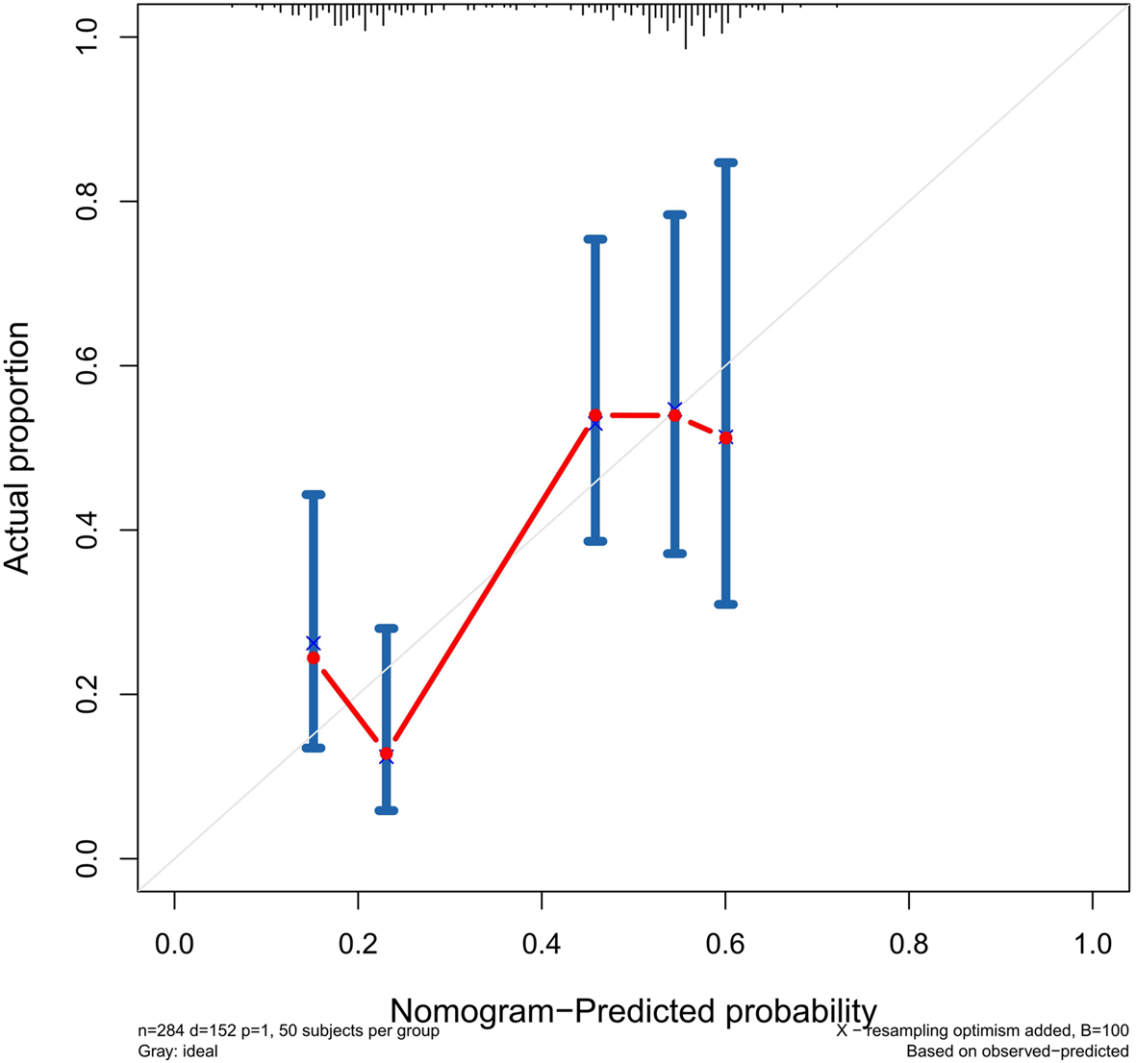
Calibration curves of external validation to predict 5-year overall survival (OS) of total gastrectomy

**Fig. 11:**
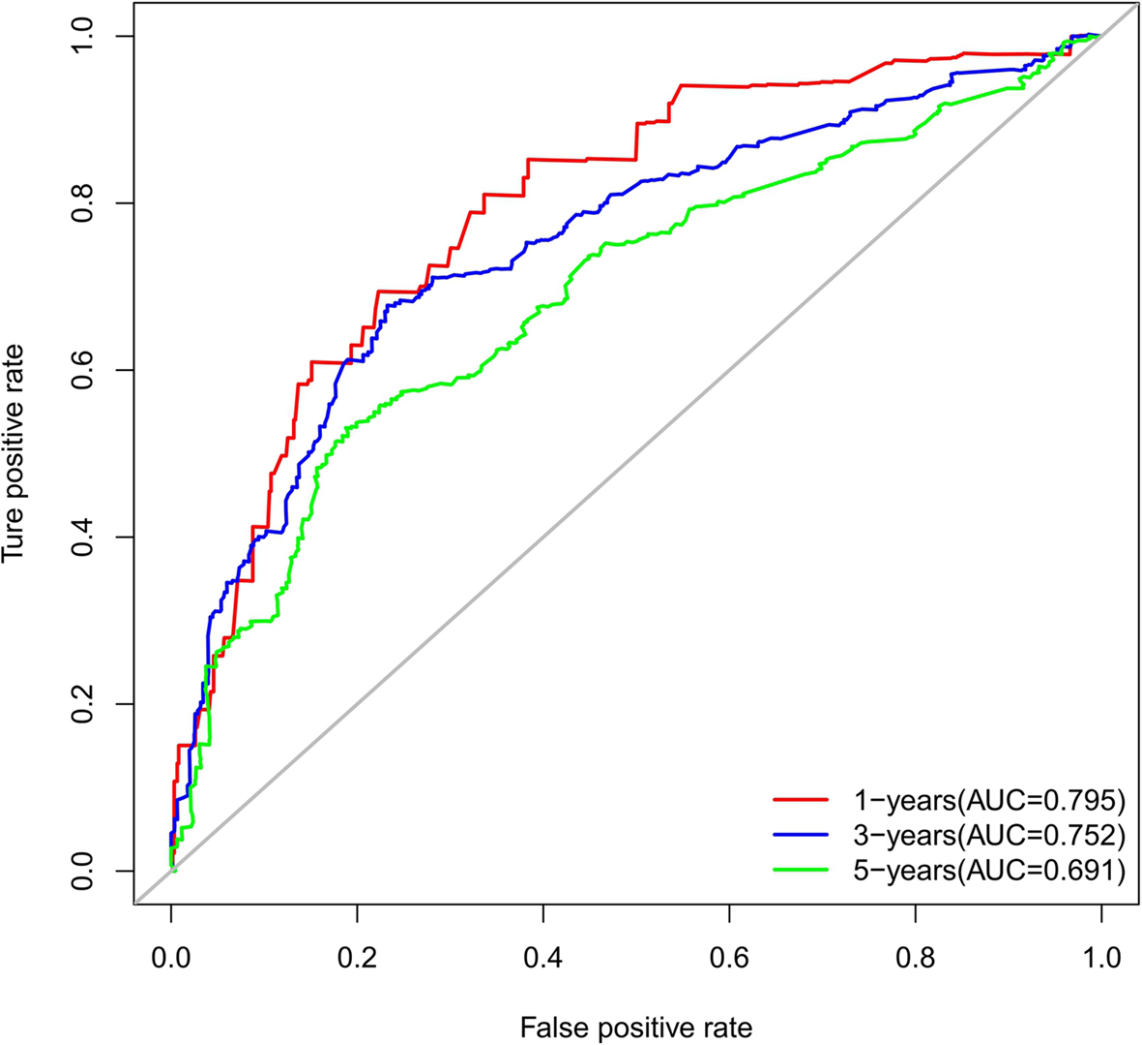
Time-dependent receiver operating characteristic (t-ROC) curves of internal validation to predict overall survival (OS) of total gastrectomy

**Fig. 12:**
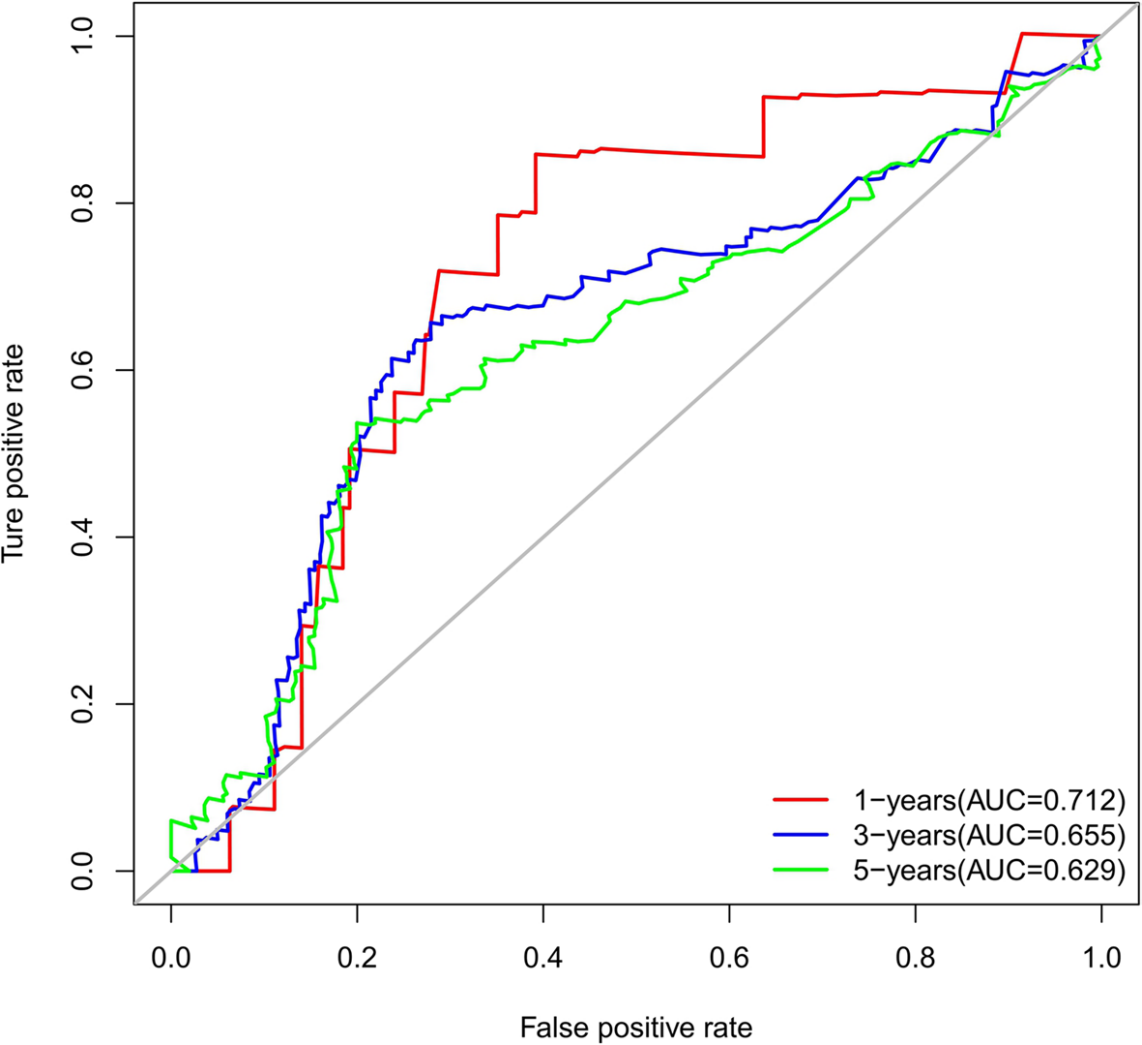
Time-dependent receiver operating characteristic (t-ROC) curves of external validation to predict overall survival (OS) of total gastrectomy

**Fig. 13:**
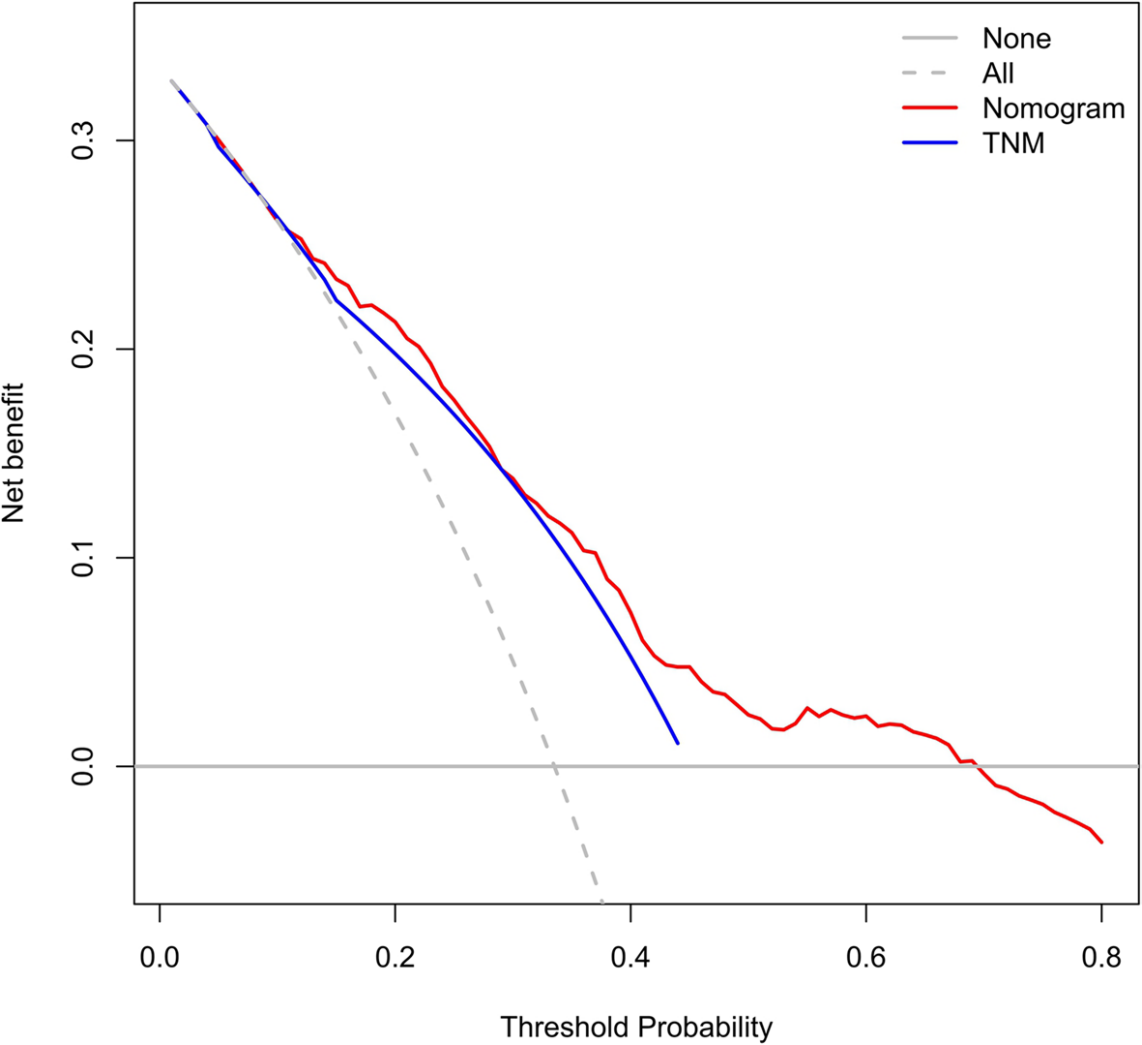
Decision curve analysis (DCA) of internal validation to predict 3-year overall survival (OS) of total gastrectomy

**Fig. 14:**
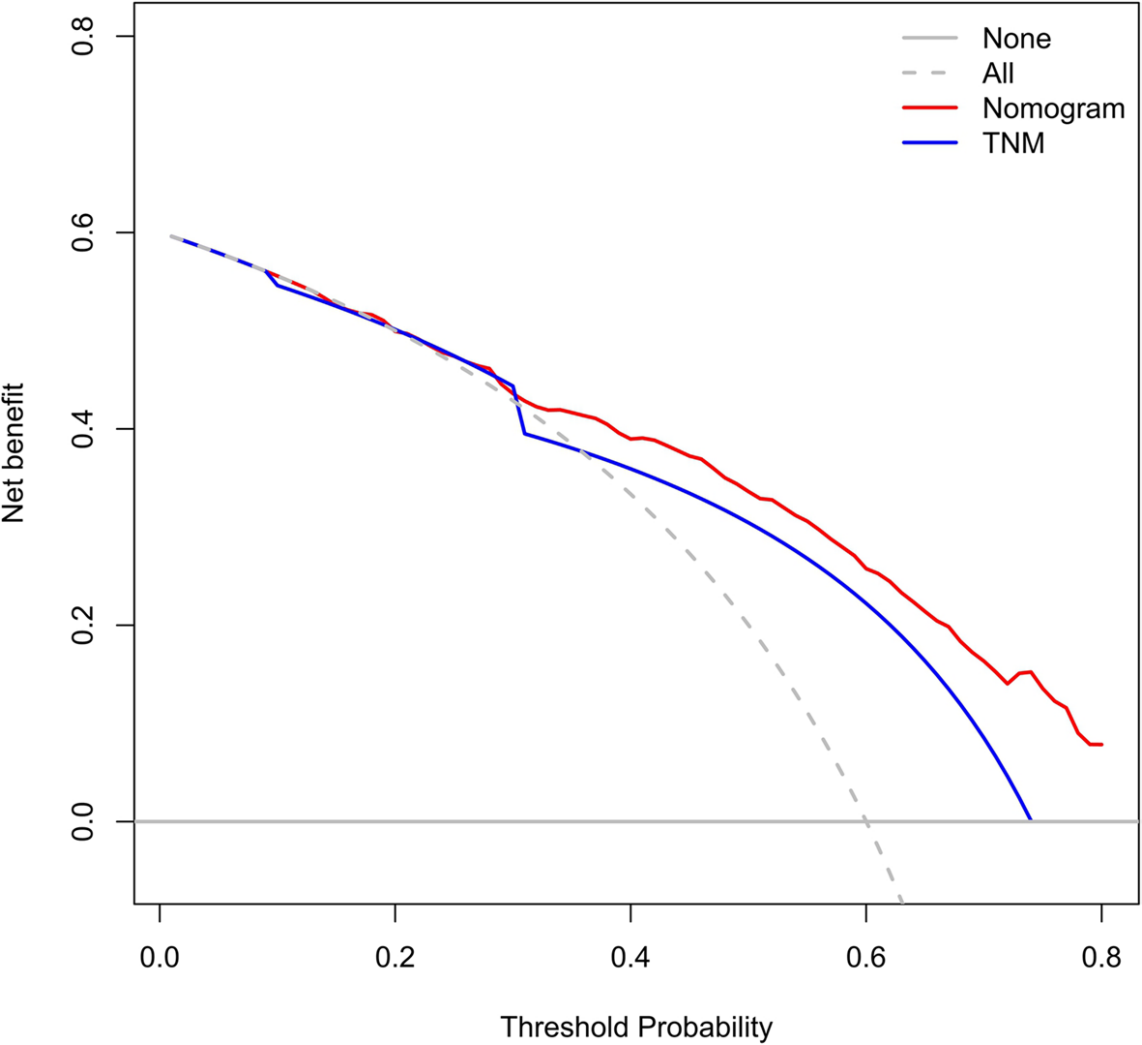
Decision curve analysis (DCA) of internal validation to predict 5-year overall survival (OS) of total gastrectomy

**Fig. 15:**
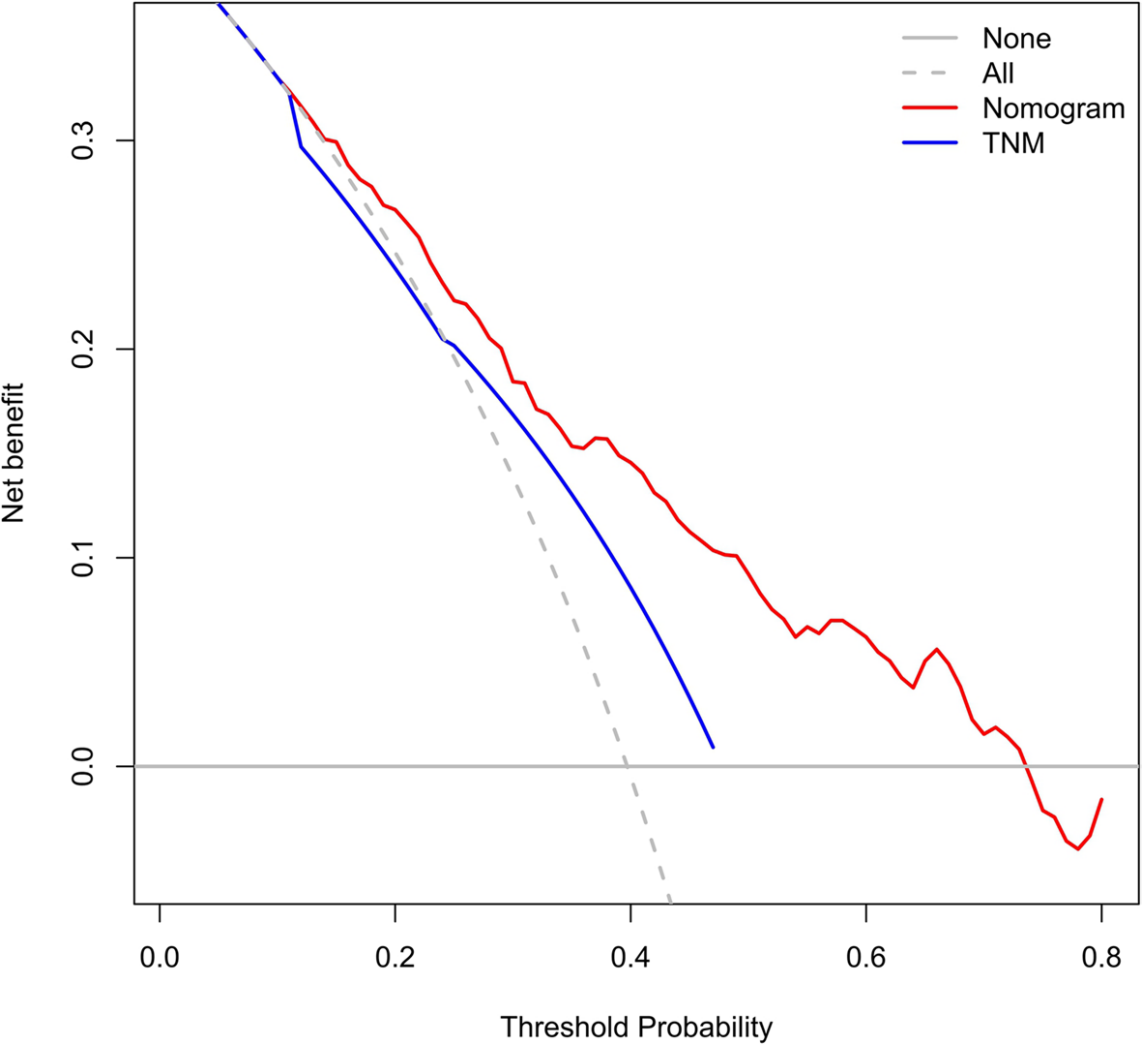
Decision curve analysis (DCA)of external validation to predict 3-year overall survival (OS) of total gastrectomy

**Fig. 16:**
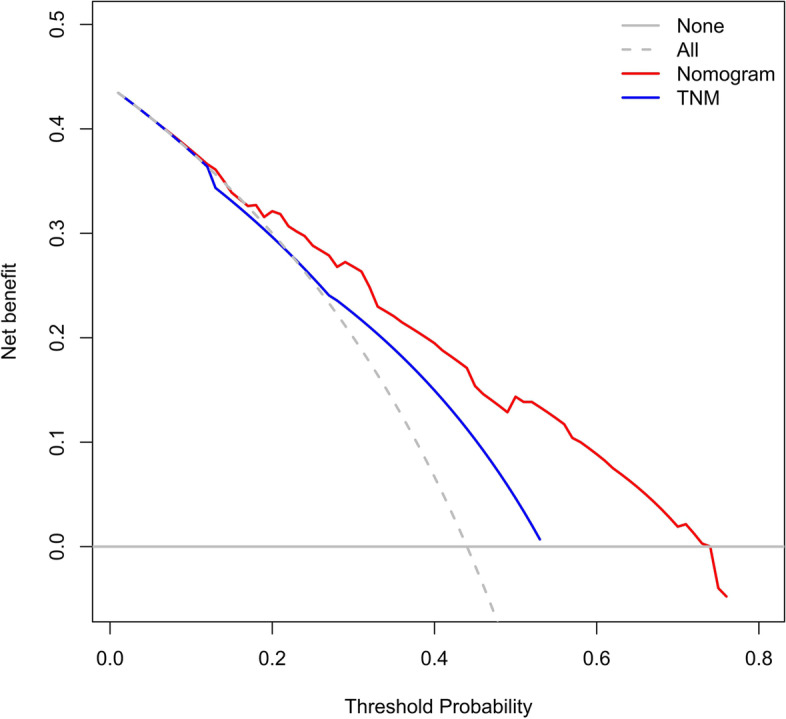
Decision curve analysis (DCA) of external validation to predict 5-year overall survival (OS) of total gastrectomy

The accuracy of this predictive nomogram was further verified using the internal validation (1000 bootstrapping from training cohort) and the external validation (from validation cohort). As shown in Figs. 5, 6, 7, 8, 9, and 10, the calibration curves for the predicted probability of 1-, 3-, and 5-year OS was consistent with the actual observations. t-ROC in the internal validation showed reliable discriminations, and the values for the area under the curve (AUC) of 1-, 3-, and 5-year OS were 0.795 (0.6672–0.8809), 0.752 (0.7370–0.8243), and 0.691 (0.6308–0.7541), respectively. In addition, the AUC values of 1-, 3-, and 5-year OS in the external validation were 0.712 (95% CI: 0.6788–0.9095), 0.740 (95% CI: 0.658–0.821), and 0.786 (95% CI: 0.688–0.884), respectively (Figs. 11 and 12). Furthermore, to evaluate the potential clinical application of our nomogram model, we conducted a DCA to compare the difference in OS between using the 8th edition of AJCC-TNM staging and using our nomogram. The DCA plots for OS discrimination ability were depicted in Figs. 13, 14, 15, and 16. The data showed that our nomogram consistently performed better than the 8th edition of AJCC-TNM staging, suggesting the potential clinical application of our model in predicting the OS of patients.

### Risk score stratification of patients with total gastrectomy

**Fig. 17:**
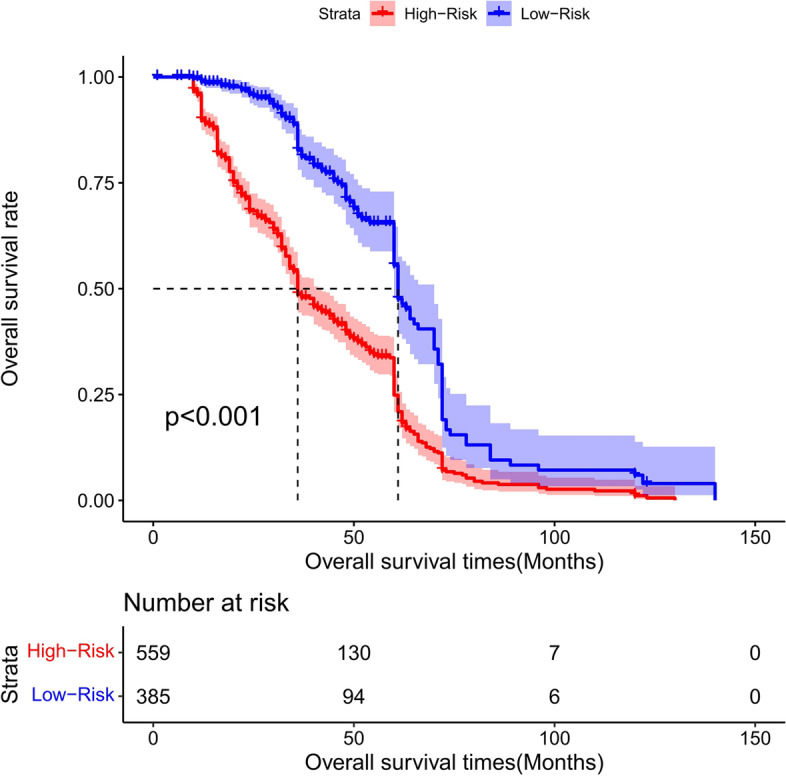
All cohort: the Kaplan–Meier survival curves for patients with different scores who underwent total gastrectomy of overall survival (OS)

**Fig. 18:**
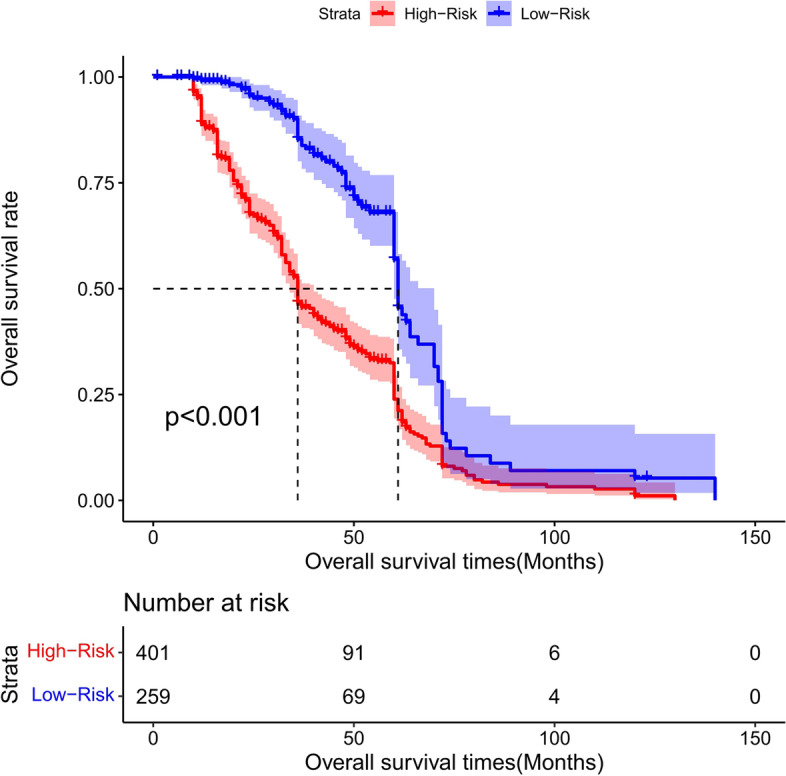
Training cohort: the Kaplan–Meier survival curves for patients with different scores who underwent total gastrectomy of overall survival (OS)

**Fig. 19:**
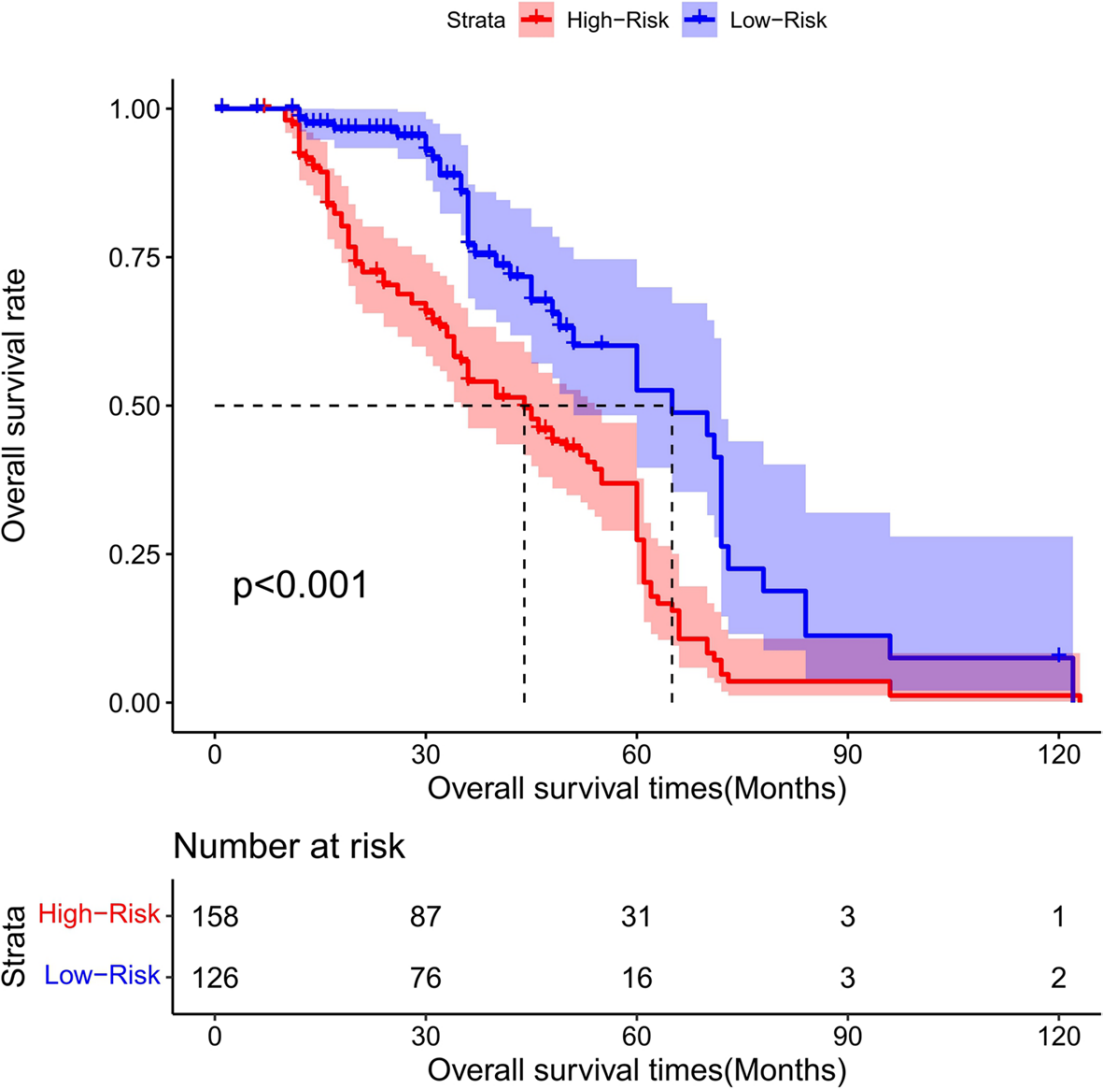
Validation cohort: the Kaplan–Meier survival curves for patients with different scores who underwent total gastrectomy of overall survival (OS)

**Fig. 20:**
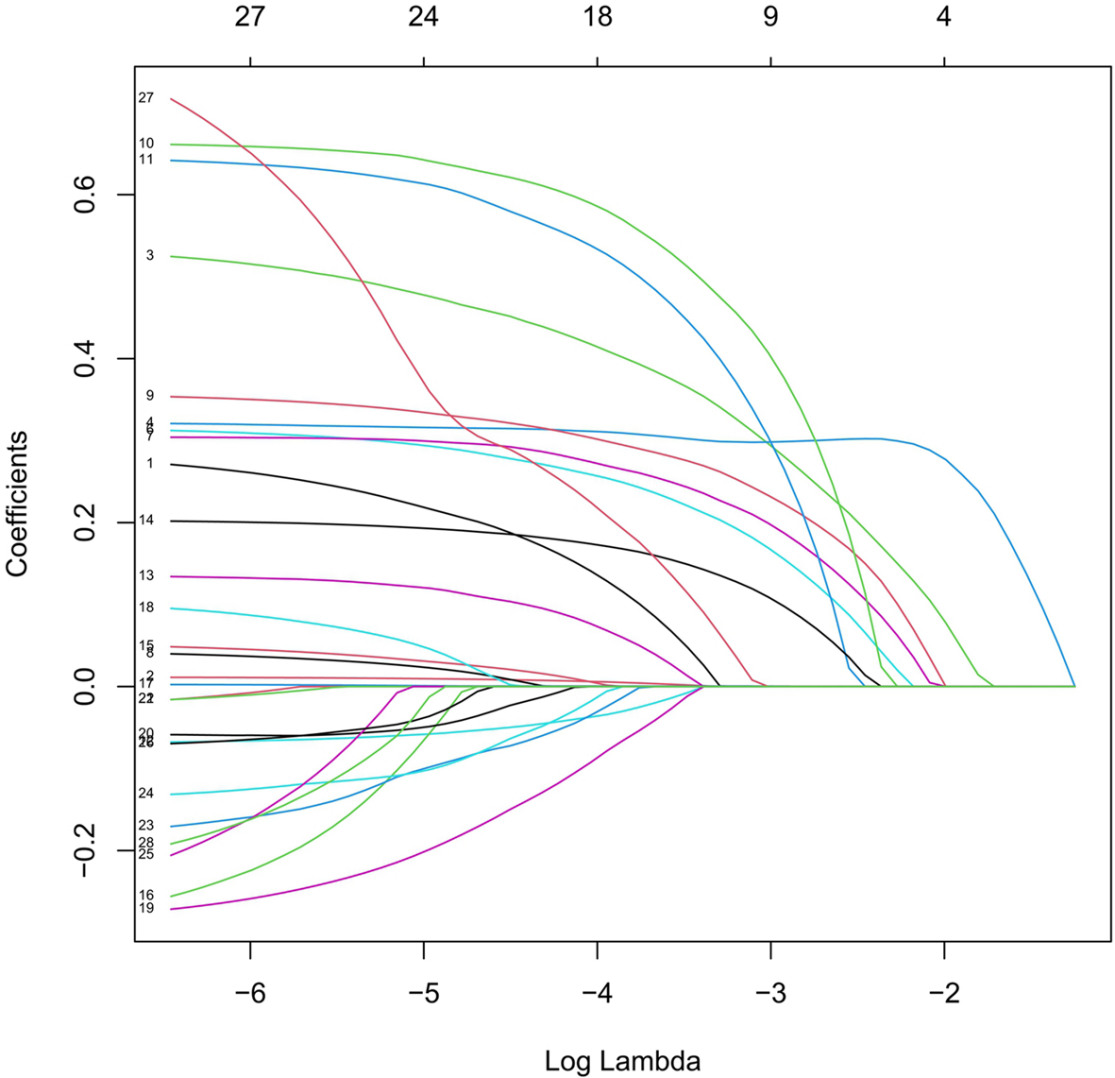
Relationship between the log lambda and the mean-squared error in the LASSO regression of progress-free survival (PFS) of total gastrectomy

The cutoff point of the total score of patients in the training group was generated by the X-tile software. According to the cutoff point of –0.370, the total patients from both the training and the validation groups were divided into 2 groups: the low- and the high-risk groups. We found that the survival probability was associated with the risk score (Figs. 17, 18, and 19). The low-risk group (total points ≤ 152.18) comprised 259 patients in the training cohort and 126 patients in the validation cohort, whereases the high-risk group (total points > 152.18) was composed of 401 patients in the training cohort and 158 patients in the validation cohort. Figure 20 showed the overall survival curves after risk score stratifications in the total patients, training cohort, and validation cohort. The *P* values in all three cohort were less than 0.001, suggesting the statistically significant difference between the low- and high-risk groups, which demonstrated that our model had an excellent risk stratification performance.

### Development and validation of the prediction model for progress-free survival (PFS)

**Fig. 21:**
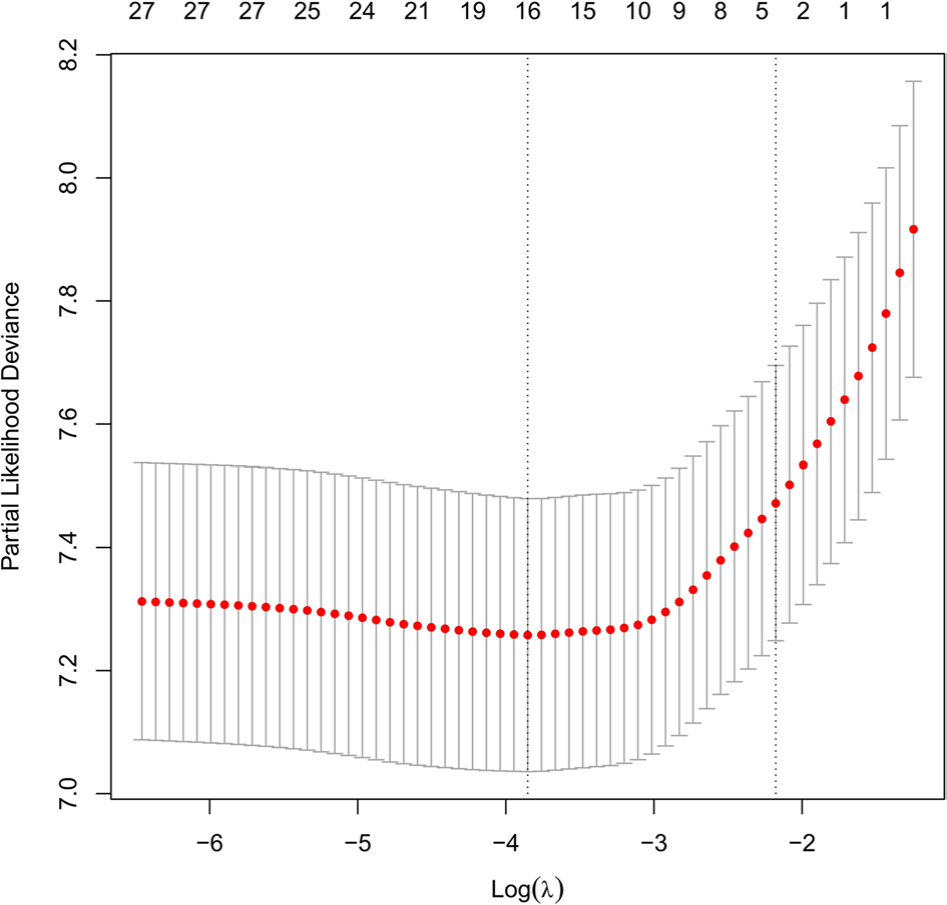
LASSO coefficient profiles of the 29 variables included in the model against the log lambda associated with progress-free survival (PFS) of total gastrectomy

**Fig. 22:**
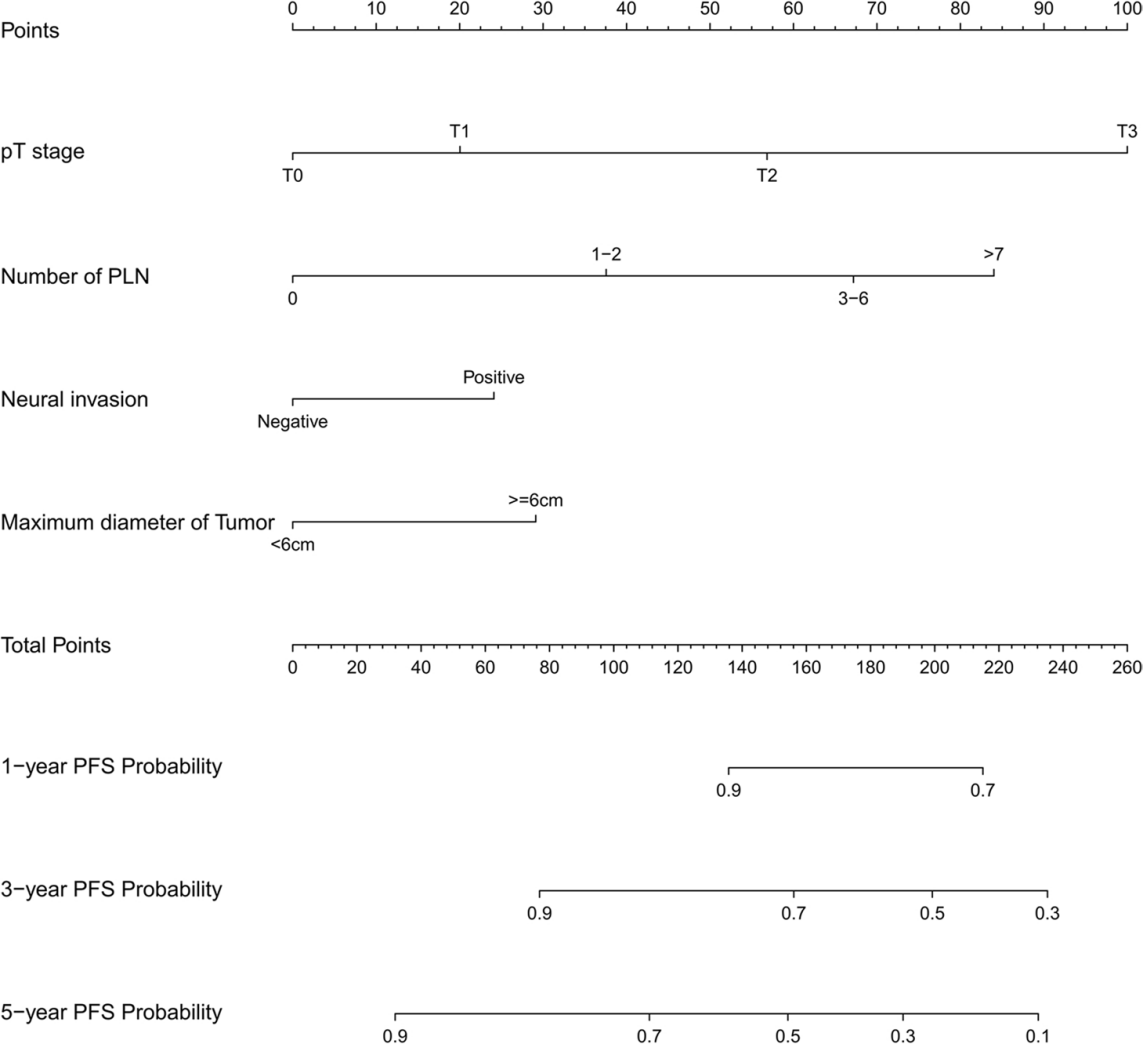
Nomogram model to predict 3-year and 5-year progress-free survival (PFS) of total gastrectomy

Furthermore, Lasso regression was carried out to identify the reliable variables to construct the prediction model for PFS. Similar to the analyses used in OS predictive model, most of the covariate coefficients shrank to zero, and only 4 remaining nonzero parameters were selected as the independent prognostic factors of the model (Figs. 20 and 21). These 4 independent factors were pT stage, number of positive lymph nodes, neural invasion, and maximum diameter of tumor. We found that the nomogram based on these four factors could predict the probability of 1-, 3-, and 5-year PFS of gastric carcinoma patients who underwent D2 + total gastrectomy (Fig. 22). This model could also be used to identify those patients with a better prognostic outcome. In the training cohort, the C-index-predicted PFS was 0.694 (95% CI: 0.654–0.713). The effectiveness of this nomogram was also compared with the discrimination by the 8th edition of AJCC-TNM staging (0.685, 95% CI: 0.635–0.751), indicating the better performance of our nomogram (Fig. 22B).

### Validation of the predictive accuracy of the nomograms for PFS

**Fig. 23:**
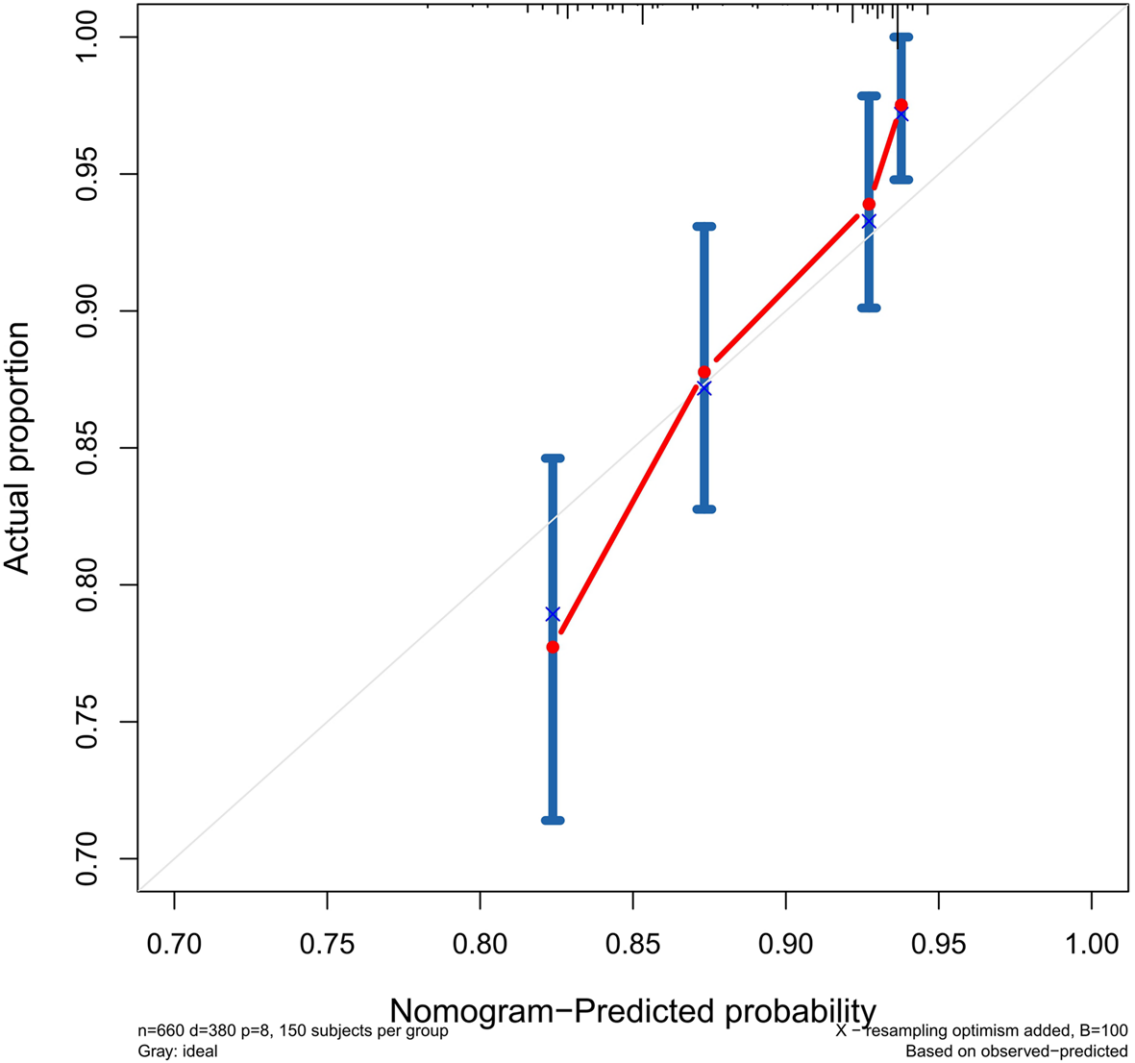
Calibration curves of internal validation to predict 1-year progress-free survival (PFS) of total gastrectomy

**Fig. 24:**
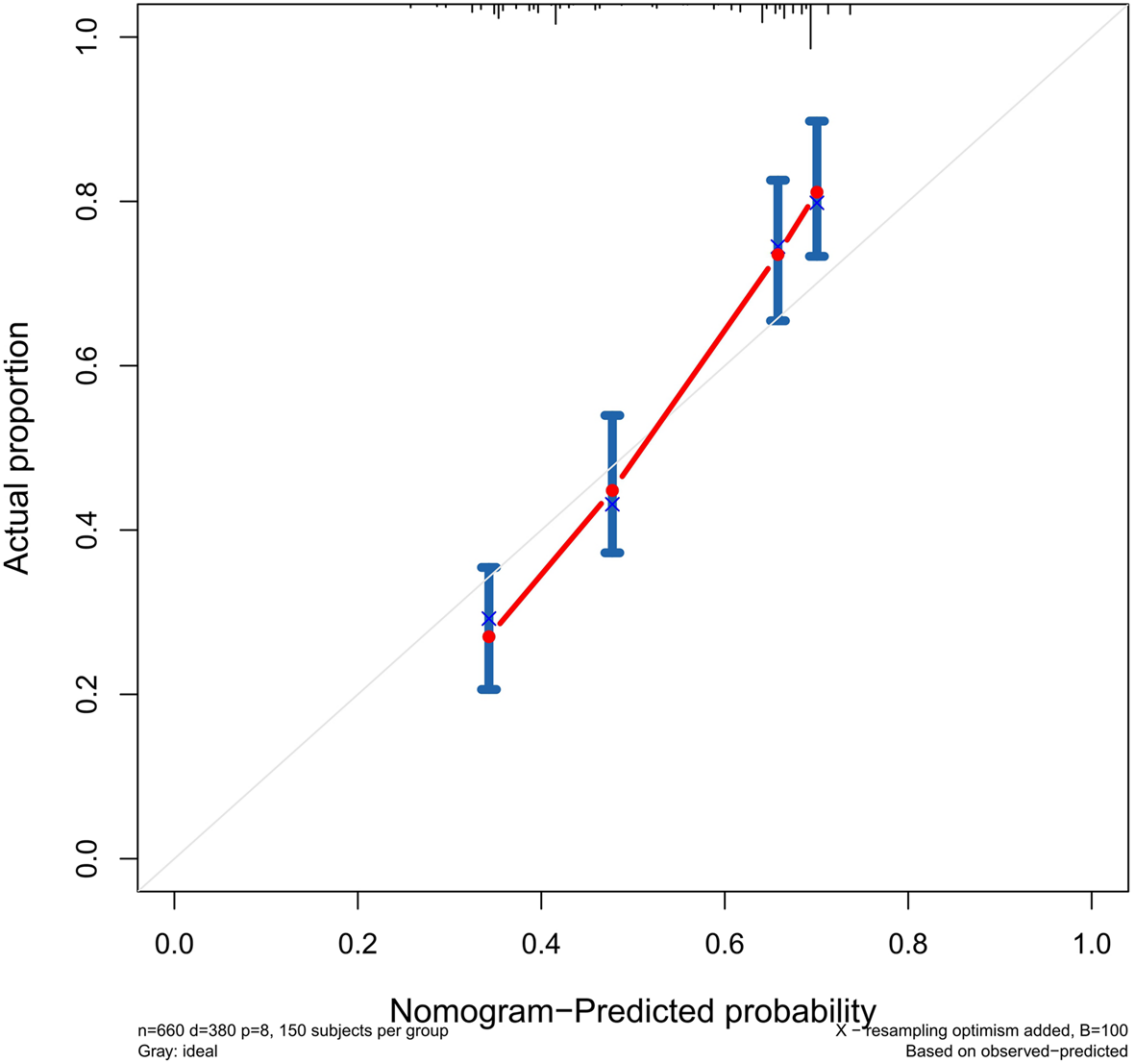
Calibration curves of internal validation to predict 3-year progress-free survival (PFS) of total gastrectomy

**Fig. 25:**
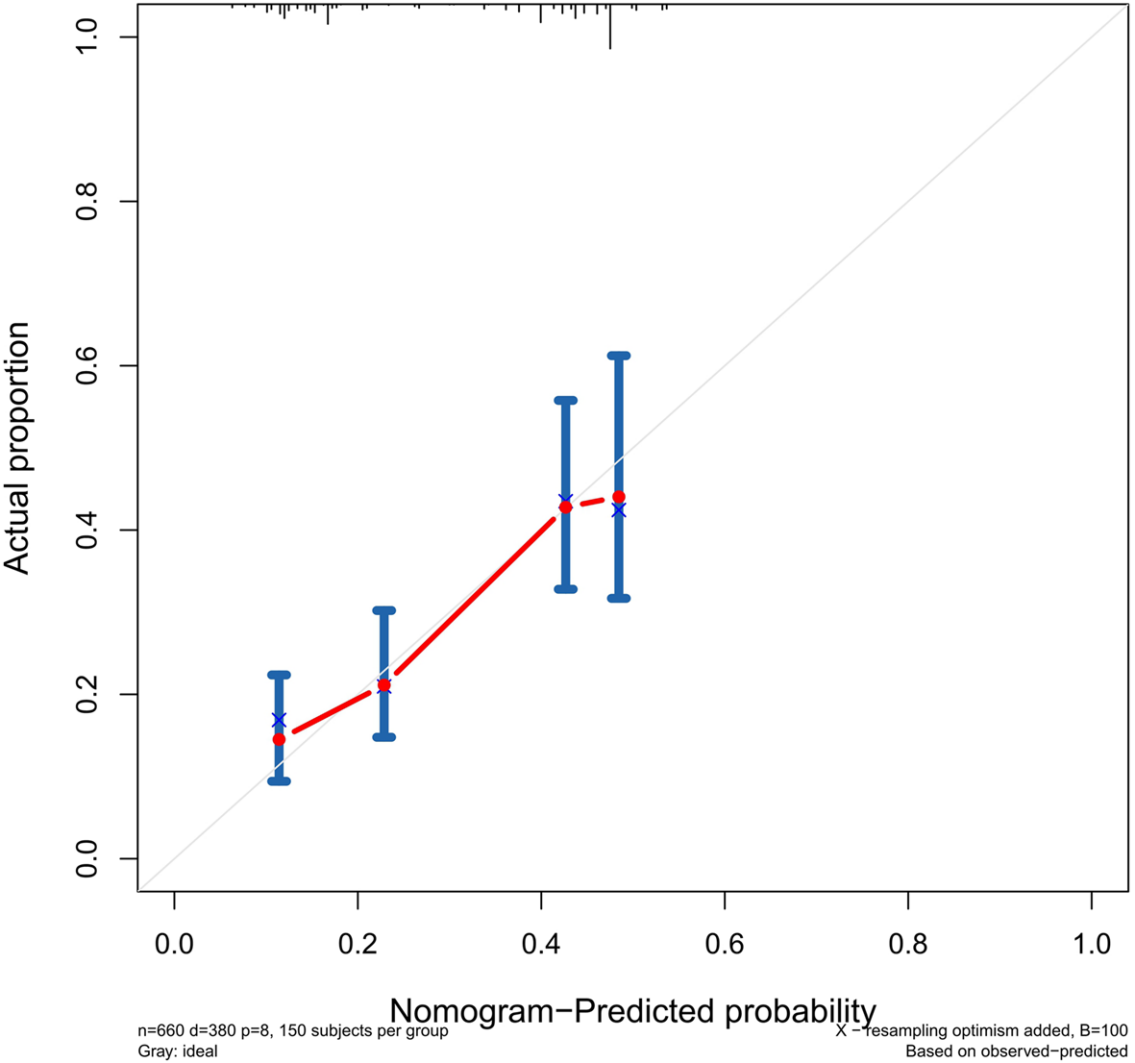
Calibration curves of internal validation to predict 5-year progress-free survival (PFS) of total gastrectomy

**Fig. 26:**
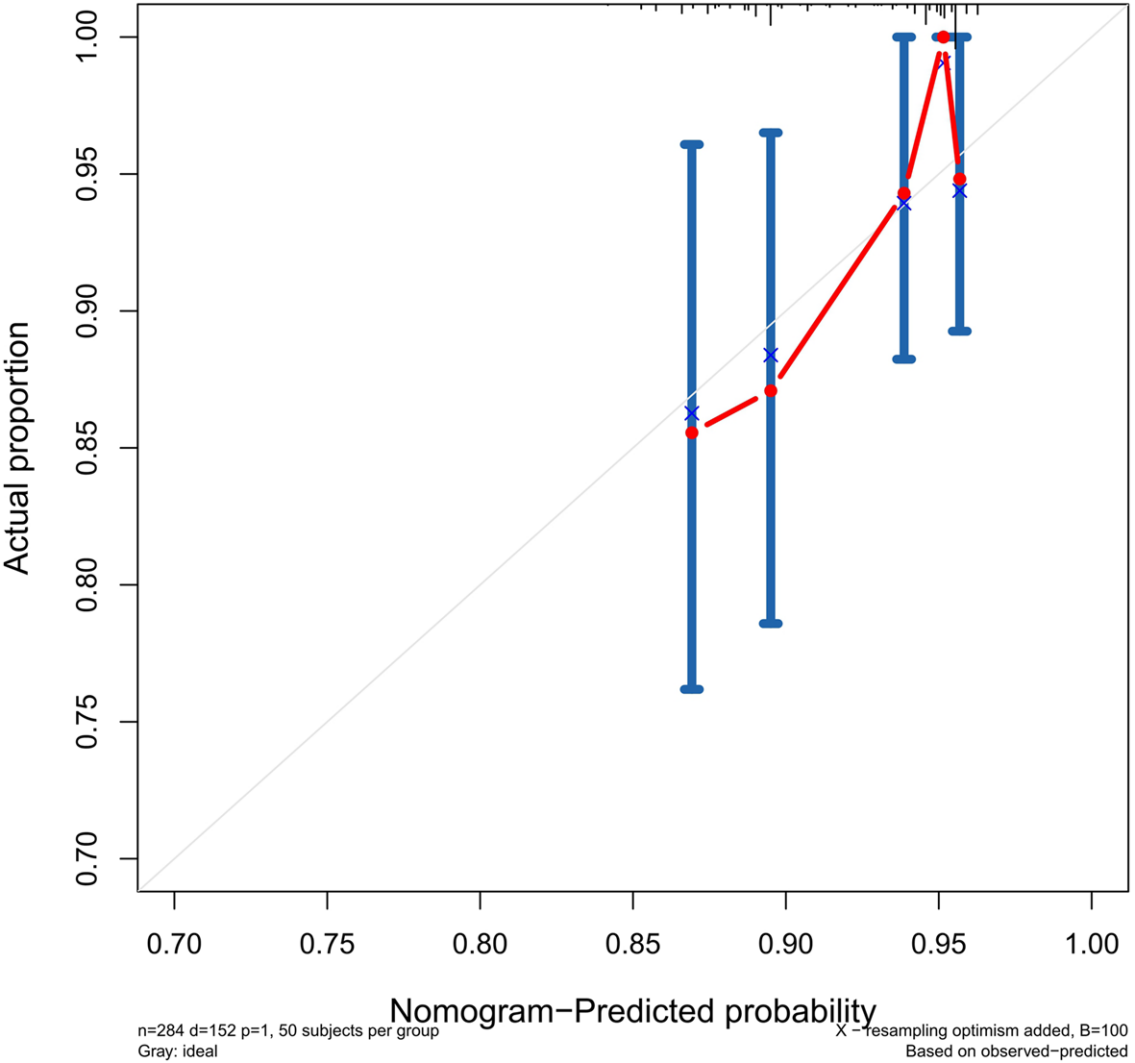
Calibration curves of external validation to predict 1-year progress-free survival (PFS) of total gastrectomy

**Fig. 27:**
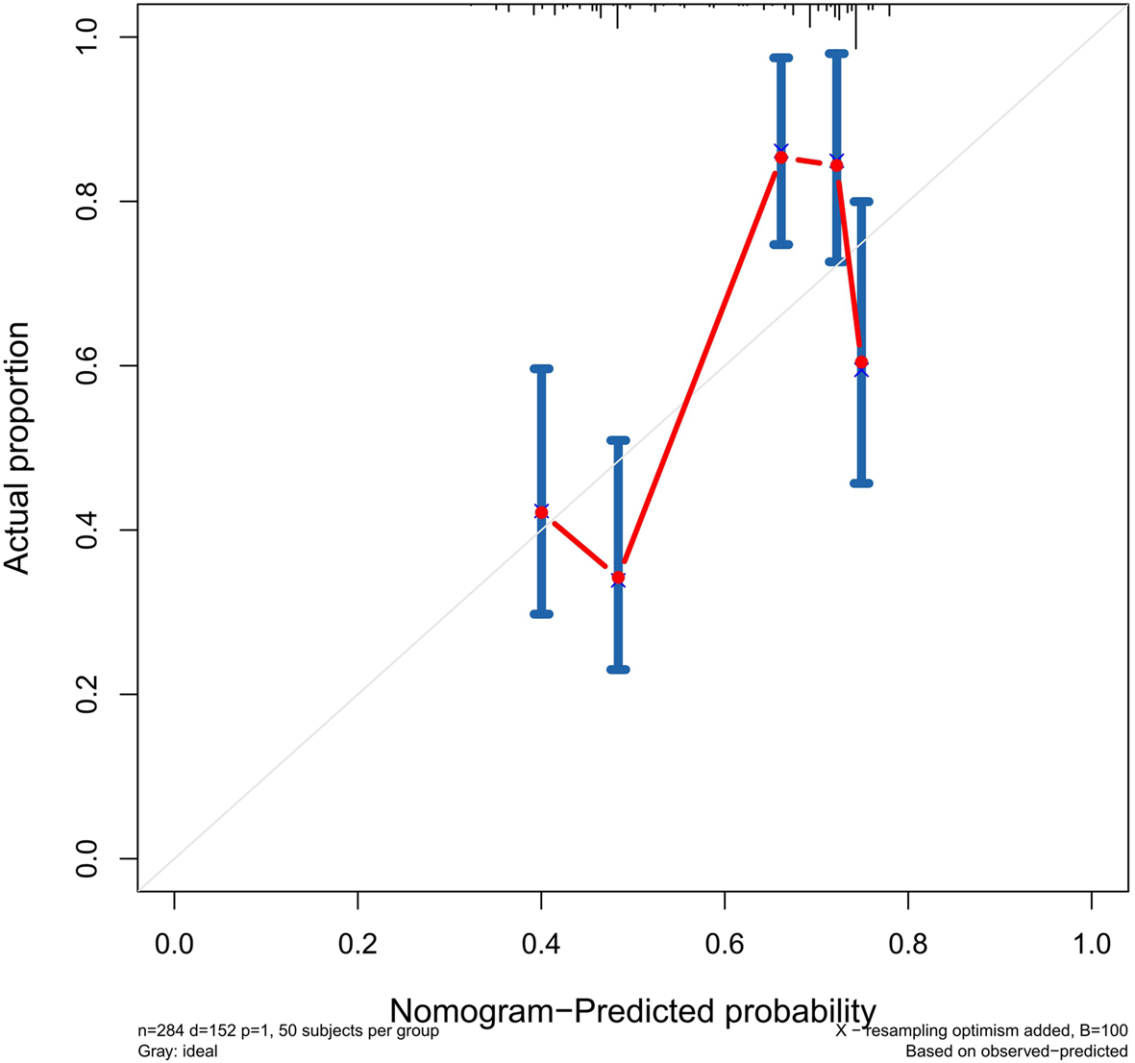
Calibration curves of external validation to predict 3-year progress-free survival (PFS) of total gastrectomy

**Fig. 28:**
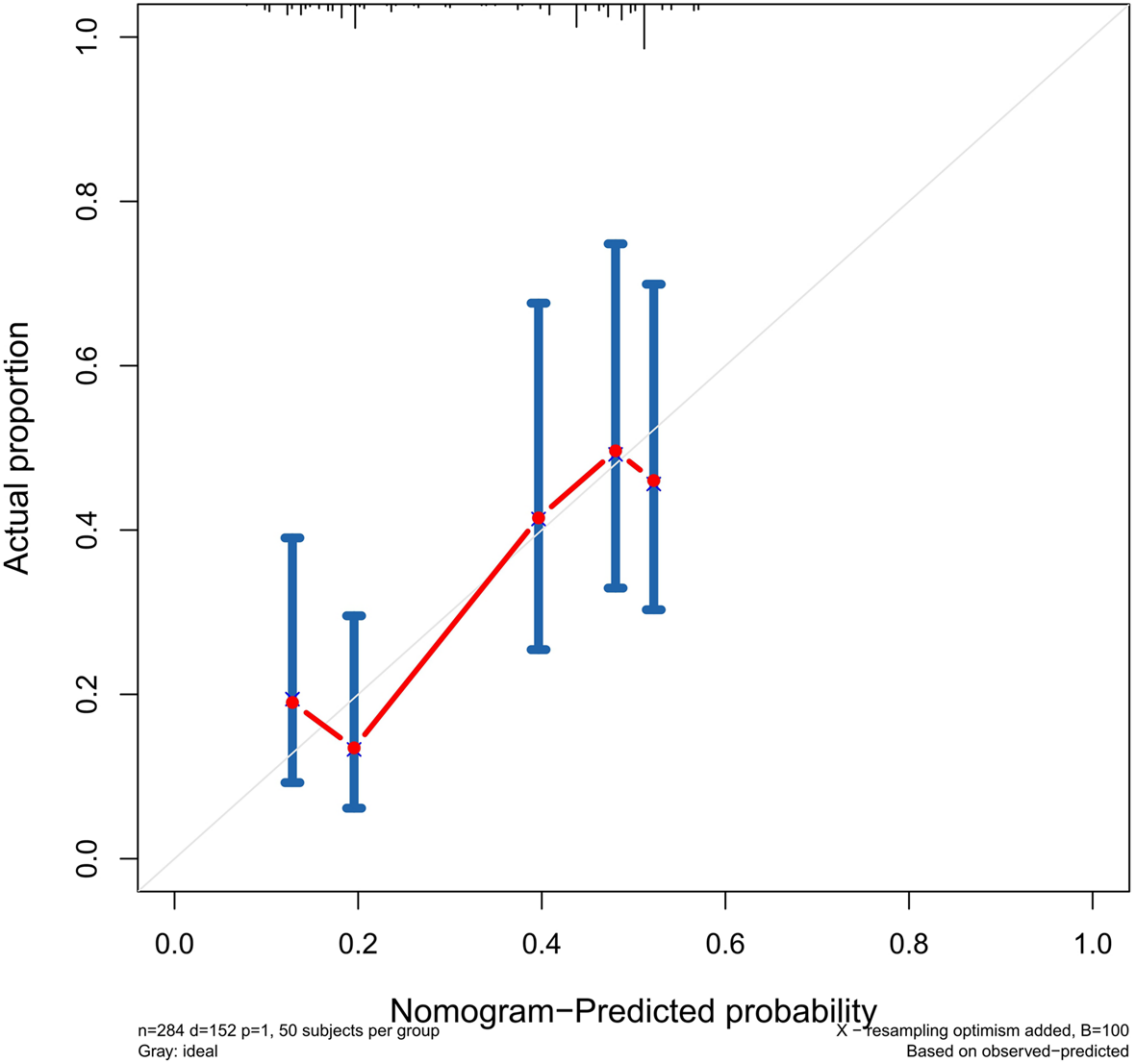
Calibration curves of external validation to predict 5-year progress-free survival (PFS) of total gastrectomy

**Fig. 29:**
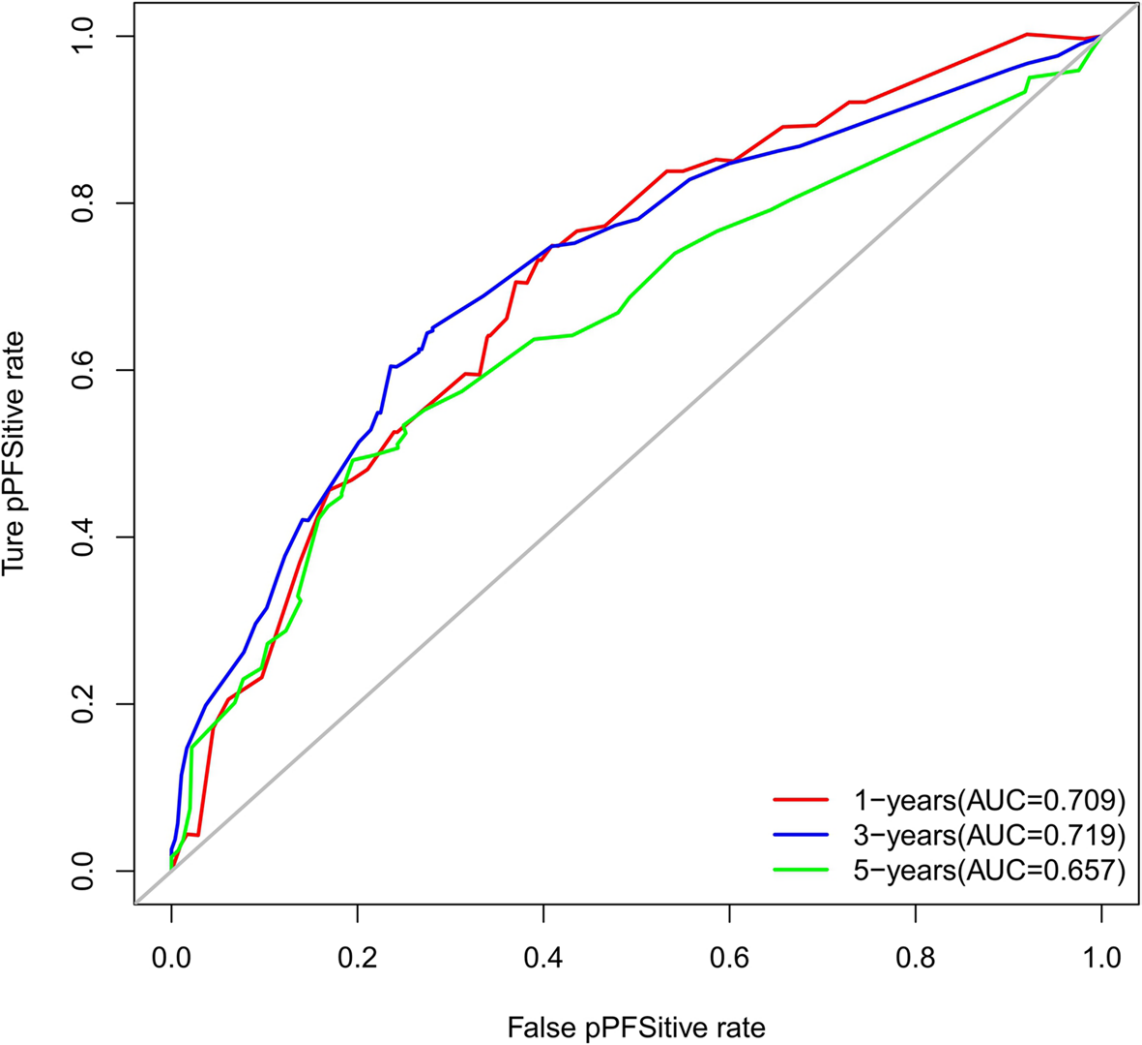
Time-dependent receiver operating characteristic (t-ROC) curves of internal validation to predict progress-free survival (PFS) of total gastrectomy

**Fig. 30:**
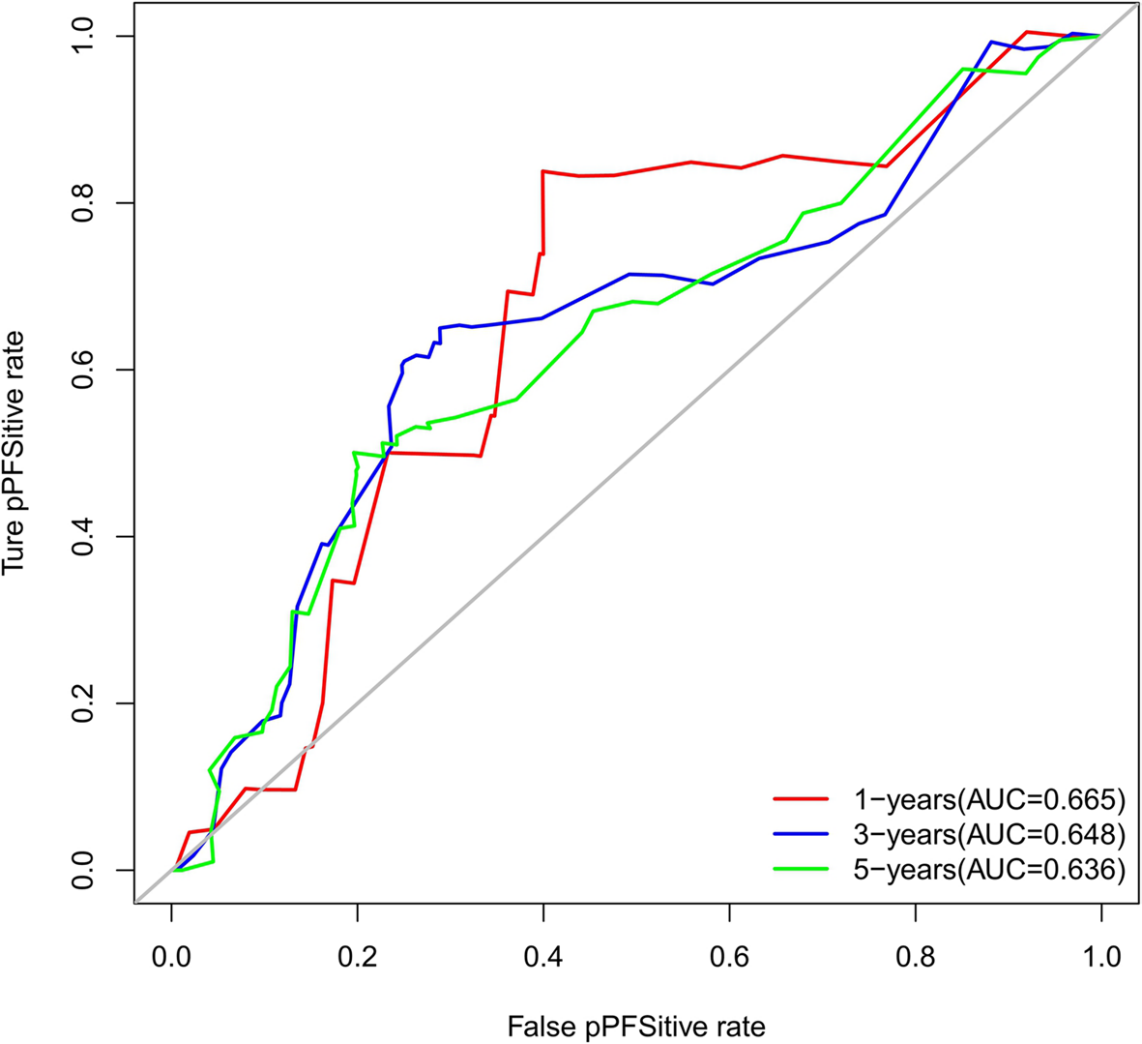
Time-dependent receiver operating characteristic (t-ROC) curves of external validation to predict progress-free survival (PFS) of total gastrectomy

**Fig. 31:**
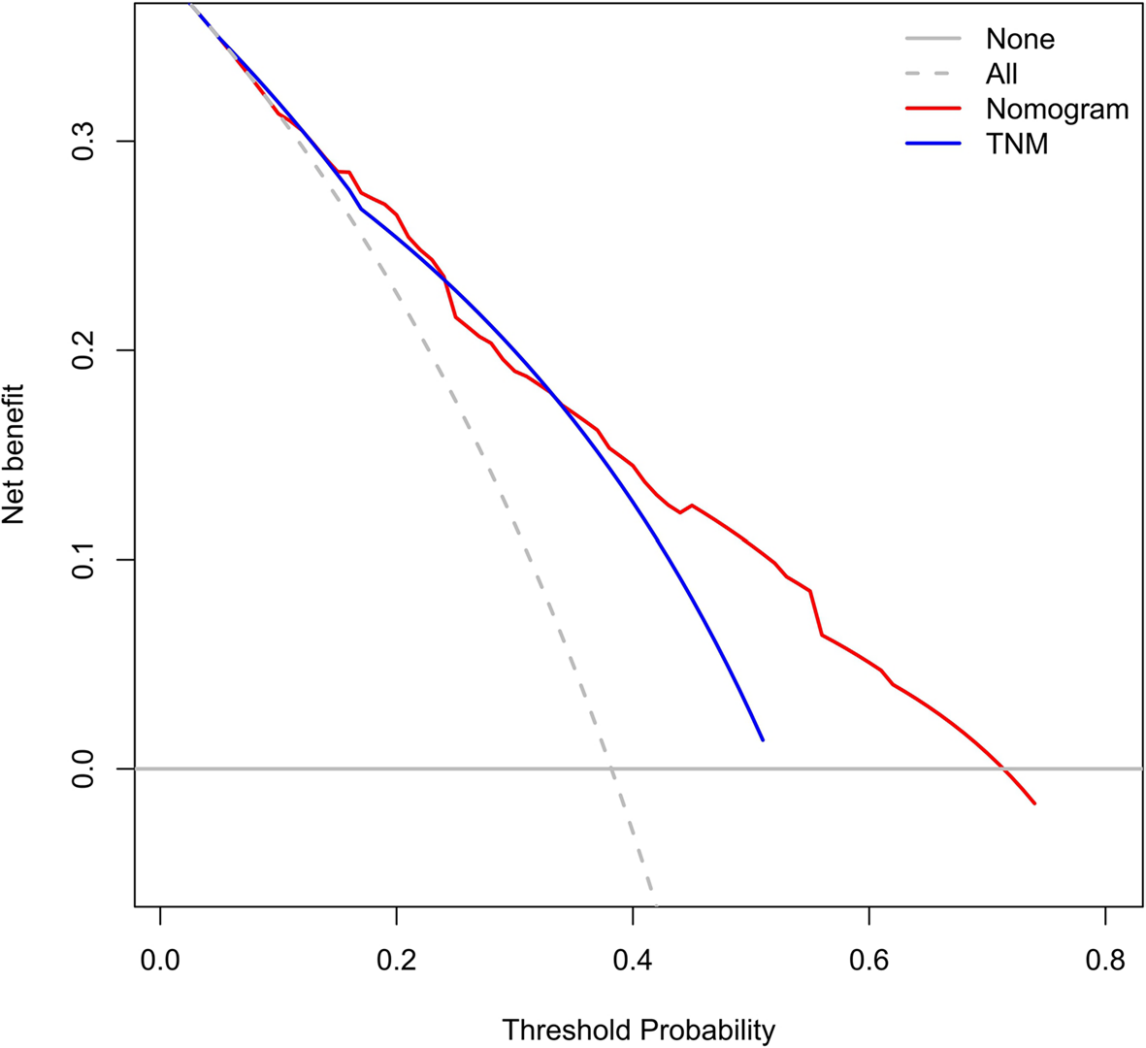
Decision curve analysis (DCA) of internal validation to 3-year progress-free survival (PFS) of total gastrectomy

**Fig. 32:**
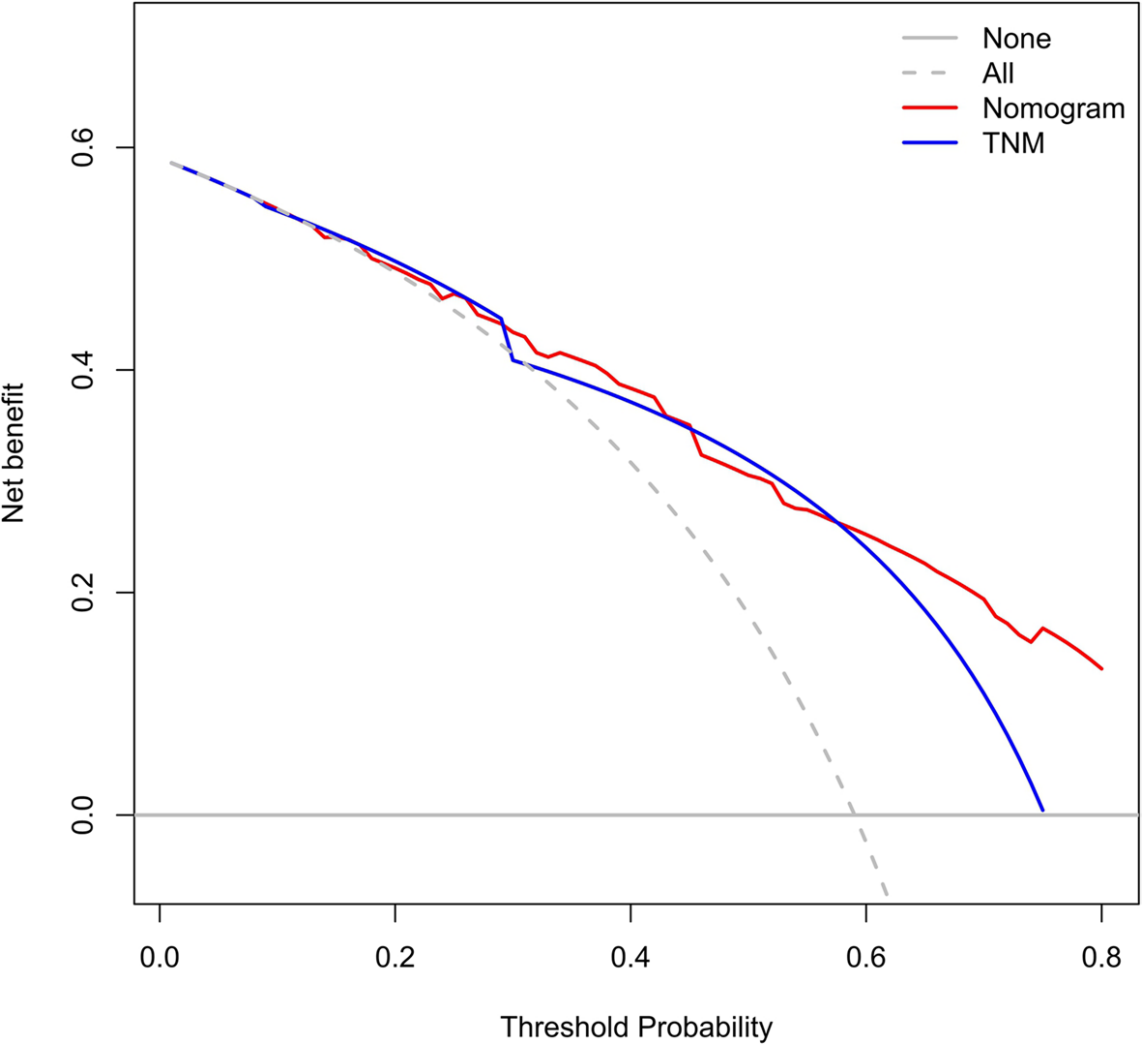
Decision curve analysis (DCA) of internal validation to predict 5-year progress-free survival (PFS) of total gastrectomy

**Fig. 33:**
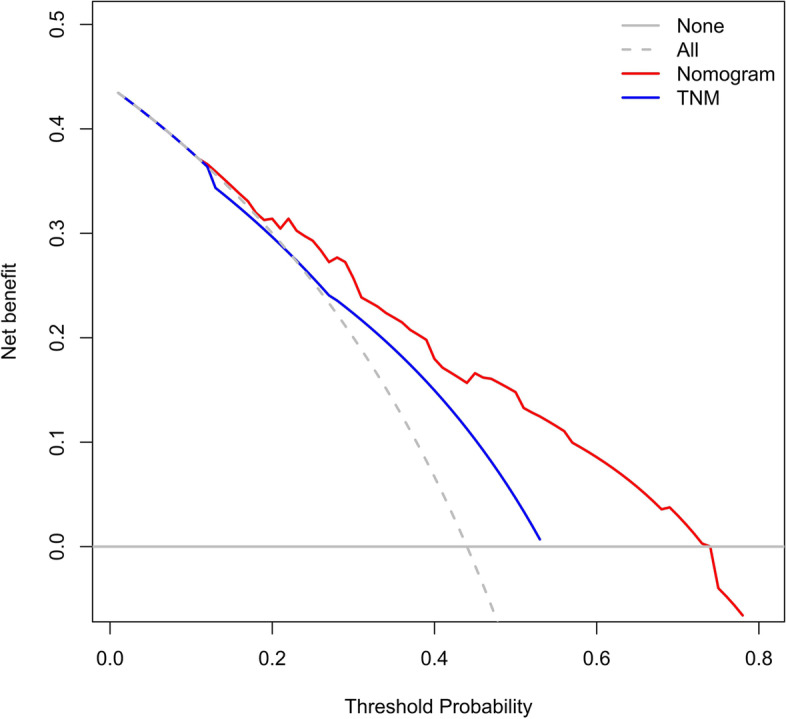
Decision curve analysis (DCA) of external validation to predict 3-year progress-free survival (PFS) of total gastrectomy

**Fig. 34:**
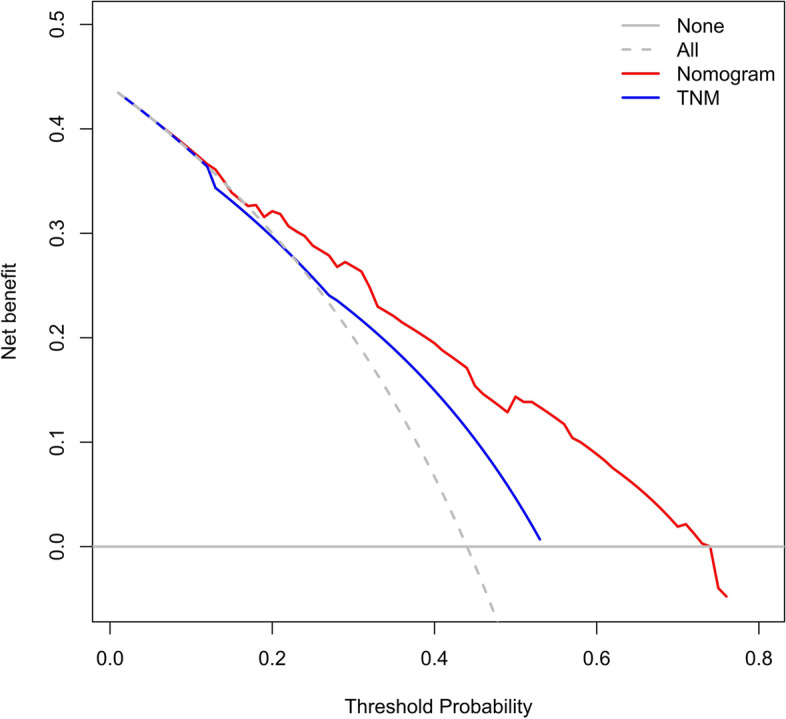
DCA of external validation to predict 5-year progress-free survival (PFS) of total gastrectomy

The accuracy of this predictive nomogram was also verified using the internal validation (1000 bootstrapping from training cohort) and the external validation (from validation cohort). As shown in Figs. 23, 24, 25, 26, 27, and 28, the calibration curves of the internal validation showed that the predictions by our nomogram were consistent with the actual observations. Furthermore, the t-ROC curve was constructed to evaluate the predictive accuracy of this nomogram. The AUC of the t-ROC curves was computed for the validation of the model’s 1-, 3-, and 5-year PFS. The AUC values for the 1-year PFS in the internal validation and the external validation were 0.709 (95% CI: 0.645–0.790) and 0.665 (95% CI: 0.474–0.800), respectively. The AUC values for the 3-year PFS in the internal validation and the external validation were 0.719 (95% CI: 0.7037–0.7974) and 0.648 (95% CI: 0.620–0.785), respectively. For 5-year PFS, the AUC values were 0.657 (internal validation, 95% CI: 0.610–0.741) and 0.636 (external validation, 95% CI: 0.528–0.739) (Figs. 29 and 30). Moreover, DCA was performed to determine the potential clinical application of our nomogram, and the DCA plots for PFS prediction were depicted in Figs. 31, 32, 33, and 34. The data demonstrated that our nomogram consistently perform better than the 8th edition of AJCC-TNM staging.

### Risk score stratification of PFS for total gastrectomy

**Fig. 35:**
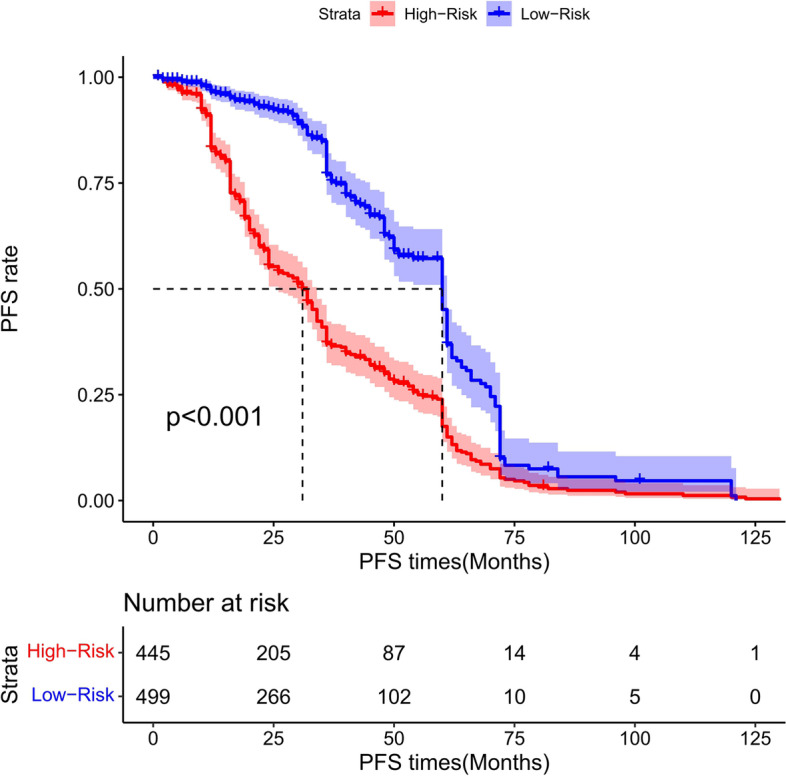
All cohort: the Kaplan–Meier survival curves for patients with different scores who underwent total gastrectomy of progress-free survival (PFS)

**Fig. 36:**
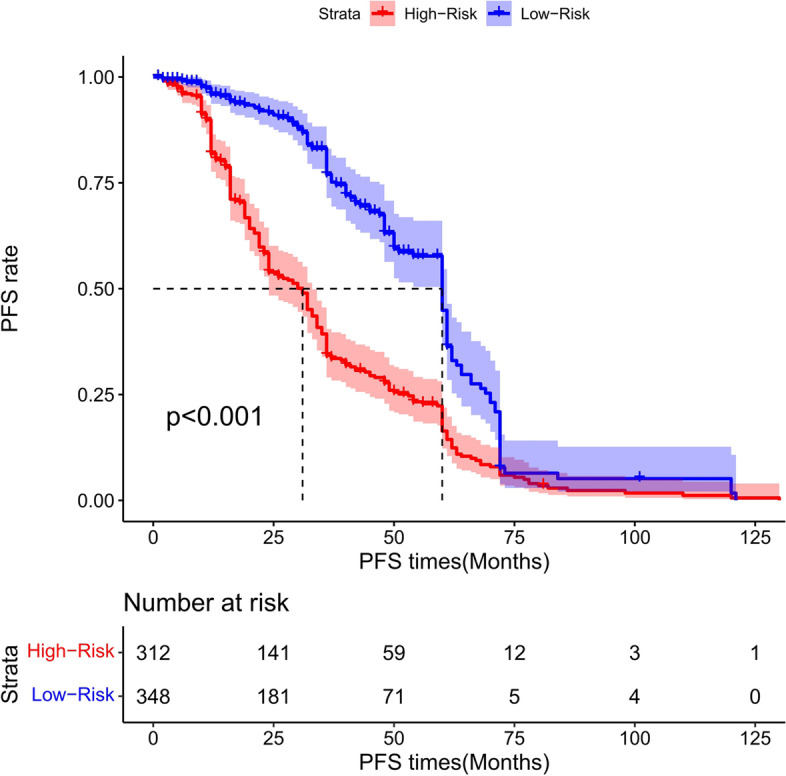
Training cohort: the Kaplan–Meier survival curves for patients with different scores who underwent total gastrectomy of progress-free survival (PFS)

**Fig. 37:**
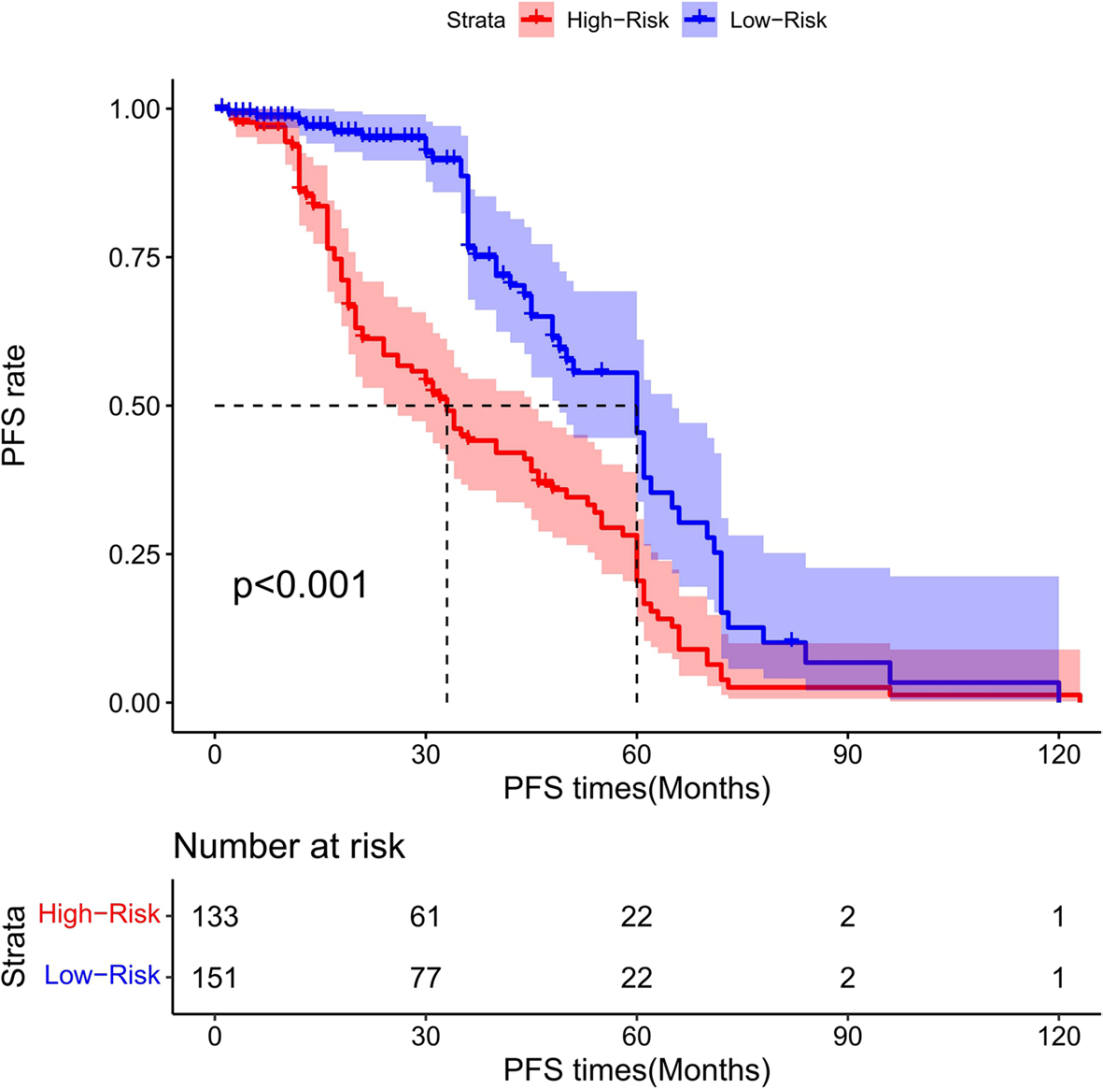
Validation cohort: the Kaplan–Meier survival curves for patients with different scores who underwent total gastrectomy of progress-free survival (PFS)

The cutoff point of the total score of PFS in the training cohort was calculated by the X-tile software. Based on the cutoff point of − 0.150, all the patients were divided into 2 groups: low- and high-risk groups. We found that the PFS probability was associated with the risk score (Figs. 35, 36, and 37). The low-risk group (total points ≤ 178.24) contained 348 patients in the training cohort and 151 patients in the validation cohort, while the high-risk group (total points > 178.24) comprised 312 patients in the training cohort and 133 patients in the validation cohort. Figure 20 showed the PFS curves after risk score stratifications in the total patient samples, the training cohort, and the validation cohort. The *P* values in the three cohorts were less than 0.001, indicating the statistically significant difference in the prognosis among the two risk stratification groups, which further demonstrated that our model had an excellent risk stratification performance.

## Discussion

The clinical studies related to total gastrectomy of gastric carcinoma are mainly focused on the anastomotic leakage and the quality of life after total gastrectomy. Esophagojejunal anastomotic leakage is regarded as the most serious complication and is responsible for the morbidity and mortality after total gastrectomy. Therefore, it is crucial to identify risk factors associated with anastomotic leakage after total gastrectomy. Nevertheless, the findings vary greatly depending on the patient population and external factors involved in the studies [14–16]. Additionally, long-term quality of life after total gastrectomy is another important area of research related to total gastrectomy, as many patients experience a series of postoperative complications such as acid reflux, gastric stasis, nausea and vomiting, eating restrictions, and diarrhea, which results in the deterioration of the long-term quality of life after total gastrectomy [9, 12, 17, 18].

Regarding to the risk stratification of total gastrectomy, a study from Japan developed and validated the preoperative risk models of morbidities associated with total gastrectomy using a Japanese web-based nationwide registry [19]. Lack of clinical information such as tumor location, intraoperative factors such as bleeding, and the extent of lymphadenectomy are the limitation of this research. Another group in Japan constructed a risk model for total gastrectomy outcomes using a nationwide internet-based database and conducted risk stratification study for total gastrectomy [20]. Distinctive molecular markers are not included in this research.

In this study, we were the first to perform a risk stratification study using the information of Chinese patients after total gastrectomy. Here, we incorporated various factors, including clinical characteristics, pathological parameters, and tumor molecular markers, to select variables by performing Lasso regression analysis and utilized Cox regression to validate the result. We finally established a reliable nomogram to predict the 1-, 3-, and 5-year OS of patients after total gastrectomy by using seven independent risk factors: pT stage, number of positive lymph nodes, vascular invasion, neural invasion, maximum diameter of tumor, Clavien–Dindo classification for complication, and Ki67 (%). Similarly, a nomogram for predicting PFS of patients with total gastrectomy was also developed by using four variables: pT stage, number of positive lymph nodes, neural invasion, and maximum diameter of tumor. Importantly, to exclude the bias that might cause differences in the prognosis of patients after radical gastrectomy in different medical centers, we evaluated the performance of our model with both internal and external validation groups. Our internal and external validation results demonstrated the satisfying performance of our model in risk prediction. In addition, we performed a population-based analysis to divide the patients into two risk groups, and the nomogram was further improved as a risk-stratifying prognosis model. This allowed us to further improve the nomogram's performance and generate a risk-stratified prognosis model, which could provide guidance to clinicians for prognosis prediction.

Currently, TNM staging is the most clinically used approach for predicting the prognostic risk; however, it has limitations in accuracy and reliability [21–23]. Several studies have shown the potential of nomograms to improve the quality of care and reduce the unnecessary tests for patients with gastric carcinoma [24]. Furthermore, many studies have explored the association between the prognosis and a variety of prognostic factors, such as age, sex, tumor size, the number of positive lymph nodes, the depth of invasion, tumor location, Lauren classification, histologic classification, and biological markers, which was subsequently used to construct various models for the prediction of prognosis [25–28].

In this study, we established two prognostic nomograms models to predict OS and PFS, respectively, and conducted risk stratification to distinguish different risk degree for patients who underwent total gastrectomy. Specifically, for patient with total gastrectomy, we calculated the risk score and performed the risk stratification of the patient. We found that the risk score was associated with the prognosis of the patient. This finding is potentially clinically useful in guiding the treatment decision-making. If the patient is predicted as low risk in OS, other postoperative treatments may not be necessary. Conversely, if the patient is predicted as high risk in OS, other combination therapies such as chemotherapy, radiotherapy, targeted therapy, and immunotherapy should be considered. Similarly, if a patient is predicted with low-risk-related PFS, suggesting a smaller chance of recurrence and mortality, it will be unnecessary for the patient to receive other postoperative treatments. However, if the patient is predicted with high-risk linked PFS, the patient will have a higher probability to experience tumor recurrence and more aggressive malignancy. Appropriate therapies should be taken not only for the treatment of tumor recurrence but also for the alleviation of the postoperative complications, including anastomotic leakage, gastrointestinal bleeding, intestinal obstruction, and nutritional complications.

Nevertheless, the current study had several limitations. First, the training and the validation cohorts used in our model construction and validation were from a single center. Validation with samples from other medical centers will be performed in the future. Second, the C-index of nomogram model for PFS was less than 0.7, and the accuracy of the nomogram related to PFS was unsatisfactory. Further investigation with larger sample size is needed. Lastly, this study did not distinguish patients between early stage and advanced stage, which may show different response to the nomograms.

## Conclusion

This study was the first to report the risk stratification and nomograms for total gastrectomy of gastric carcinoma for Chinese population. Combination therapies are recommended for patients in the high-risk score group, but not for the low-risk group patients. In addition, treatment for various postoperative complications and tumor recurrence are recommended to improve the PFS of patients in high-risk group.

## Data Availability

The raw datasets generated during the current study are available from the corresponding author on reasonable request. All data analyzed during this study are included in this published article; Yifan Li is willing to share my data in this article.

## Abbreviations

OS: Overall survival
PFS: Progress-free survival
C-index: Concordance index
t-ROC: Time-dependent receiver operating characteristic
DCA: Decision curve analysis
AUC: Area under the curve
